# The Paradoxical Role of Cellular Senescence in Cancer

**DOI:** 10.3389/fcell.2021.722205

**Published:** 2021-08-12

**Authors:** Jing Yang, Mengmeng Liu, Dongchun Hong, Musheng Zeng, Xing Zhang

**Affiliations:** ^1^Melanoma and Sarcoma Medical Oncology Unit, State Key Laboratory of Oncology in South China, Collaborative Innovation Center for Cancer Medicine, Sun Yat-sen University Cancer Center, Guangzhou, China; ^2^State Key Laboratory of Oncology in South China, Collaborative Innovation Center for Cancer Medicine, Sun Yat-sen University Cancer Center, Guangzhou, China

**Keywords:** cancer, senescence, aging, senescence-associated secretory phenotype, senescent cell

## Abstract

Cellular senescence occurs in proliferating cells as a consequence of various triggers including telomere shortening, DNA damage, and inappropriate expression of oncogenes. The senescent state is accompanied by failure to reenter the cell cycle under mitotic stimulation, resistance to cell death and enhanced secretory phenotype. A growing number of studies have convincingly demonstrated a paradoxical role for spontaneous senescence and therapy-induced senescence (TIS), that senescence may involve both cancer prevention and cancer aggressiveness. Cellular senescence was initially described as a physiological suppressor mechanism of tumor cells, because cancer development requires cell proliferation. However, there is growing evidence that senescent cells may contribute to oncogenesis, partly in a senescence-associated secretory phenotype (SASP)-dependent manner. On the one hand, SASP prevents cell division and promotes immune clearance of damaged cells, thereby avoiding tumor development. On the other hand, SASP contributes to tumor progression and relapse through creating an immunosuppressive environment. In this review, we performed a review to summarize both bright and dark sides of senescence in cancer, and the strategies to handle senescence in cancer therapy were also discussed.

## Introduction

The cellular senescence was first described by Hayflick and colleagues in which they observed that after serial cultivation *in vitro*, normal human fibroblasts exhausted their capacity to divide and entered a state of irreversible growth arrest, whereas cancer cells did not enter this growth arrest state (Hayflick and Moorhead, 1961; Hayflick, 1965). After years of debate as to whether it is an artifact *in vitro* culture or an important biological process, senescence is now considered to be an important biological mechanism involved in tumorigenesis (Loaiza and Demaria, 2016). Cellular senescence, a stable state of cell cycle arrest, occurs in proliferating cells as a consequence of various triggers including telomere shortening, DNA damage, and inappropriate expression of oncogenes (Robles and Adami, 1998; von Zglinicki and Martin-Ruiz, 2005; Courtois-Cox et al., 2008; d’Adda di Fagagna, 2008; Romagosa et al., 2011). The unrelenting shortening of telomeres during cellular proliferation and the accumulation of DNA damage are the basis of senescence, which cause a permanent arrest of cell cycle as a strategy to prevent genomic instability.

There is mounting evidence that the accumulation of senescent cells can contribute to organismal aging, and might involve cancer prevention (Braig et al., 2005; Chen et al., 2005). In addition to decreasing replicative capacity, activation of senescence in different contexts and tissues leads to increased expression of inflammatory cytokines that elicit immune-mediated tumor clearance (Xue et al., 2007; Kang et al., 2011). However, studies over the past decades have convincingly demonstrated a paradoxical role for senescence emanating from its special secretory profile. Recent studies challenge the conventional view, showing that senescence can counterintuitively promote cancer stemness and aggressiveness (Dou and Berger, 2018; Milanovic et al., 2018).

Cancer treatment has traditionally relied on cytotoxic strategies, assuming that complete destruction of cancer cells can optimize the survival of patients. It is increasingly recognized that achieving complete cell death within a solid tumor based on this theory, may cause severe side effects to patients (Ewald et al., 2010). Inducing cytostasis which permanently decreases the replicative capacity of cells without inducing cancer cell death has been considered as a new weapon for cancer therapy (Ewald et al., 2010). Recent studies utilizing cytostatic treatments have reported promising preliminary results, suggesting that therapy-induced senescence (TIS), a promising approach to induce cytostasis, may be effective in preventing tumor growth (Dorr et al., 2013; Baell et al., 2018). On the contrary, some literatures have reported that TIS plays a negative role in the treatment of cancer. Here, we summarized the causes and the controversial roles of senescence in cancer, and the strategies to handle senescence in cancer therapy were also discussed.

## Triggers of Cellular Senescence

**FIGURE 1:**
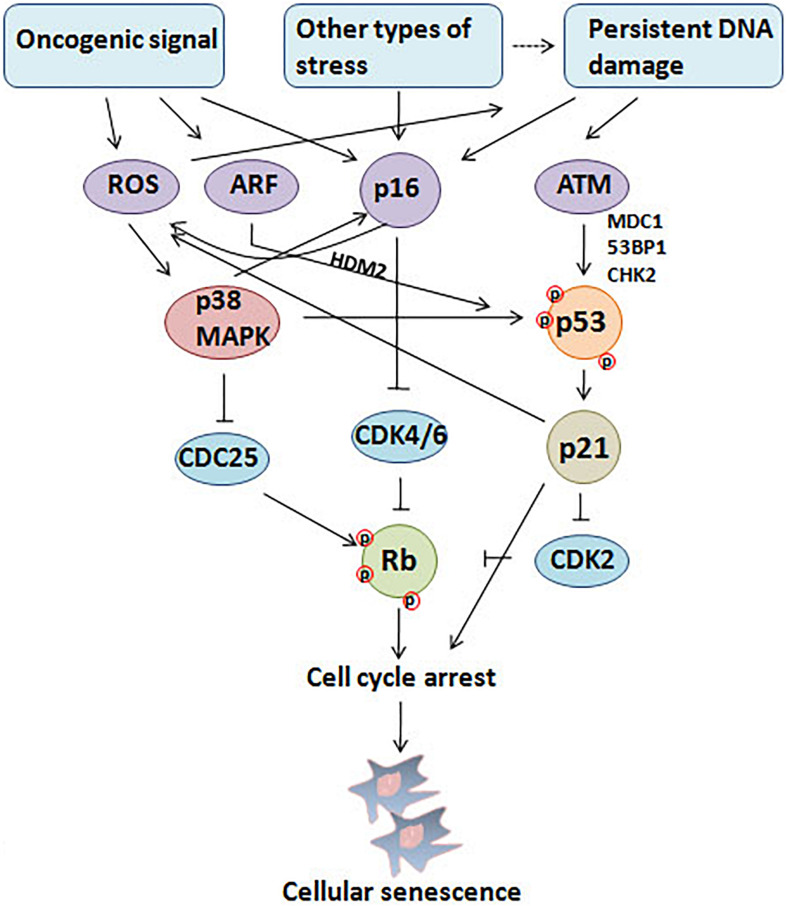
Triggers and main effector pathways of senescence. Persistent DNA damage induced by diverse stimuli and abnormal oncogenic signal lead to senescence mainly by regulating p16INK4a–pRb and p53/p21 pathways.

**FIGURE 2:**
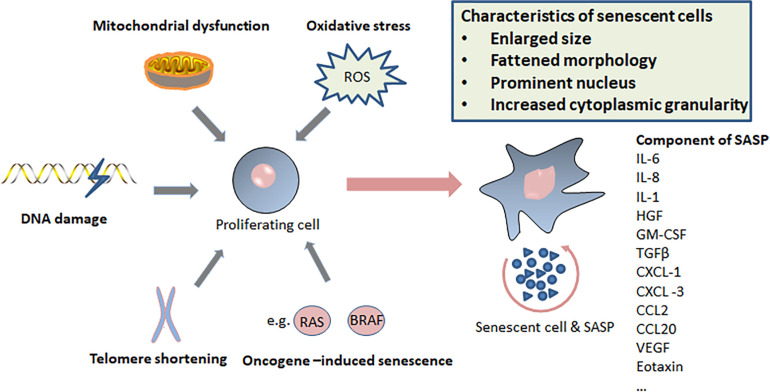
Triggers of senescence, characteristics of senescent cells, and components of senescence-induced secretory phenotype (SASP).

Cellular senescence is considered to be a stress response triggered by multiple mechanisms, such as DNA damage, telomere shortening, oncogene activation, tumor suppressor loss, centrosome dysfunction, and epigenomic damage (Ben-Porath and Weinberg, 2005; Rodier et al., 2011; Schleich et al., 2020; Wu et al., 2020a). These mechanisms are increasingly well-understood at the molecular level. The main effect pathways and triggers of senescence are shown in the Figures 1, 2.

### DNA Damage

Persistent DNA Damage Response (DDR) is considered to be a common mechanism that is crucial to the establishment and maintenance of senescence phenotypes (d’Adda di Fagagna, 2008). Breakages of sugar-phosphate DNA backbone are potent activators of DDR, which can cause the double-stranded DNA breaks (DSBs) and the exposure of single-stranded DNA (d’Adda di Fagagna, 2008). DSBs can be induced by ionizing radiation, topoisomerase inhibitors and many other agents. Oxidative stress and several DNA-damaging agents often induce DNA base damage and single-strand breaks (Robles and Adami, 1998; Chang et al., 2002; Parrinello et al., 2003; Sedelnikova et al., 2004; Barascu et al., 2012). DSBs can result in increased secretion of inflammatory cytokines, such as interleukin-6 (IL-6); however, this occurs only after the establishment of persistent DNA damage signaling rather than after transient DDRs (Rodier et al., 2009). In order to initiate and maintain this cytokine response, protein Ataxia-Telangiectasia Mutated (ATM), Nijmegen breakage syndrome protein 1 (NBS1) and Checkpoint kinase 2 (CHK2) are required, but the cell-cycle arrest enforcers p53 and pRb are not necessary (Rodier et al., 2009).

Ataxia telangiectasia-mutated (ATM), a member of the phosphoinositide-3 kinase-like kinase (PIKK) family, is the chief transducer of the DSBs signal. Single-stranded DNA is sensed by ataxia telangiectasia and Rad3-related (ATR) which is the primary mediator of ultraviolet (UV) light damage and stalled replication forks (Shiloh, 2006; Zou, 2007). The activation of the DNA damage checkpoint depends on the concerted activities of the upstream DNA damage kinases ATM and ATR, the downstream protein kinases CHK1 and CHK2, and DDR proteins 53BP1, mediator of DNA damage checkpoint protein 1 (MDC1), and NBS1 (d’Adda di Fagagna et al., 2003; Lou et al., 2003; Shiloh, 2003). Senescence initiated by DNA damage always relies on p53, usually accompanied by the expression of p21 (Di Leonardo et al., 1994; Campisi and d’Adda di Fagagna, 2007). In many cells, DNA damage can also induce the expression of p16, which provides a second barrier to prevent the growth of cells with severe DNA damage (Stein et al., 1999; Beausejour et al., 2003).

The DDR can also induce inflammation and senescence by inhibiting the autophagy of GATA4 (Kang et al., 2015). Normally, GATA4 is degraded by p62-dependent selective autophagy (Kang et al., 2015). During cellular senescence, ATM and ATR block the degradation of GATA4 (Kang et al., 2015). In turn, GATA4 induces the activation of transcription factor NF-κB and the initiation of senescence-associated secretory phenotype (SASP), therefore facilitating senescence (Kang et al., 2015). Notably, this process is completely independent from p53 and p16INK4a.

Most evidence of aging-related DNA damage comes from experiments with high doses of environmental gene toxins. To construct a model of spontaneous DNA damage *in vivo*, ERCC1-/Δ mice (with reduced expression of ERCC1-XPF endonuclease) with impaired capacity to repair the nuclear genome were bred (Robinson et al., 2018). This study demonstrated that spontaneous endogenous nuclear DNA damage is sufficient to drive an increase in ROS levels and lead to an accelerated accumulation of senescent cells *in vivo* (Robinson et al., 2018). Furthermore, when spontaneous endogenous DNA damage is the primary injury to a mammalian system, cellular senescence and ROS abundance are increased, leading to further damage and senescence (Robinson et al., 2018). On the other hand, DNA damage can induce a transient proliferation arrest, allowing cells to repair their damage (Kuilman et al., 2010). The DDR allows cells to sense damaged DNA and respond by arresting cell-cycle and repairing the DNA damage, if possible (Campisi and d’Adda di Fagagna, 2007). However, when DNA damage exceeds a certain threshold and cannot be repaired, such as with an uncapped telomere, cells are destined to undergo apoptosis or senescence (Campisi and d’Adda di Fagagna, 2007).

### Telomere Shortening

Telomeres are complexes of proteins and nucleotides of TTAGGG repeats, which cap the ends of linear chromosomes (Meyne et al., 1989; Wright et al., 1997). It has been shown that some telomere-associated factors can inhibit DDR activity, for example, telomeric repeat-binding factor 2 (TRF2; a double-stranded telomeric DNA-binding protein), and protection of telomeres 1 (PoT1; a single-stranded telomeric-binding protein) can inhibit the activity of ATM and ATR, respectively (Karlseder et al., 2004; Denchi and de Lange, 2007). The key role of telomere-related factors has been confirmed by observations that stripping TRF2 or POT1 from telomere DNA can trigger an obvious DDR (d’Adda di Fagagna, 2008).

Due to the mechanisms of replication and the inability to completely replicate the ends of linear DNA molecules, telomeres shorten as each cell division (Hayflick, 1965; Levy et al., 1992; Allsopp et al., 1995). Therefore, telomeres act as “molecular clocks,” reflecting the replicative history of a primary cell. The enzyme telomerase is able to re-elongate the ends of chromosomes, thus preventing telomere shortening (Maicher et al., 2012). In the majority of human somatic cells, telomeres are shortened due to insufficient telomerase expression (Maicher et al., 2012). In normal cells, telomere shortening results in an “uncapped” telomere, which triggers DDR and ultimately leads to irreversible cellular senescence (Bodnar et al., 1998; d’Adda di Fagagna et al., 2003). Loss of the G-rich strand and the association of nuclear foci of γ-H2AX (a phosphorylated form of the histone variant H2AX) with telomeres have been found in the senescence of normal human fibroblasts (d’Adda di Fagagna et al., 2003; Stewart et al., 2003). Recently, inappropriate DNA:cytoplasm ratio resulted from excessive enlargement of cells has also been reported to contribute to senescence (Xu et al., 2015). Furthermore, the activation of p53 and p21 is up-regulated during senescence, most of which contain one or more γ-H2AX foci; p53, p21 and RB act in a linear genetic pathway in the regulation of cell entry into senescence (Wei et al., 2003; Herbig et al., 2004).

Since it is triggered by telomere shortening caused by repeated cell divisions, this process is termed replicative senescence. Replicative senescence can be bypassed by the ectopic expression of the catalytic subunit of the telomerase holoenzyme (hTERT), confirming its dependence on telomere shortening (Bodnar et al., 1998; Vaziri and Benchimol, 1998). Telomere attrition limits the proliferative lifespan of many human cells and induces cells to undergo replicative senescence (Harley et al., 1990).

### Oncogene-Induced Senescence

Oncogene-induced senescence (OIS) is a powerful and persistent anti-proliferative response caused by down-regulation of tumor suppressor genes or over-expression or mutation of oncogenes (Courtois-Cox et al., 2008). Cellular senescence-related genes were summarized in the Table 1. Unlike replicative senescence, OIS cannot be bypassed by expression of hTERT, which confirms that OIS is independent from telomere dysfunction (Wei et al., 1999). The oncogene used in the original description of OIS is RAS; early studies have reported that expression of oncogenic RAS (HRAS^V12^) in primary human or rodent cells causes a permanent G1 arrest and promotes premature senescence by activating p53 and p16 (Serrano et al., 1997; Lin et al., 1998). This process involves the activation of the MAPK cascade, which is identical to RAS-induced mitogenesis in immortal cells (Franza et al., 1986; Khosravi-Far et al., 1995; White et al., 1995; Urano et al., 1996; Lin et al., 1998). The biological outcomes (cycle arrest or forced mitogenesis) of MAPK activation largely depend on the context and the integrity of the senescence machinery (Lin et al., 1998). Previous findings strongly support the view that normal cells possess fail-safe mechanisms to limit the effects of RAS mitogenic signaling, and the safeguards involve p53 and p16 (Serrano et al., 1997; Lin et al., 1998). Furthermore, NORE1A (a member of the RASSF family) can activate OIS as a barrier against RAS-mediated transformation (Barnoud et al., 2017).

**TABLE 1:** Cellular senescence-related gene.

Senescence-related genes	Up- or down-regulation	Cancer type	References
Ras	Up	Colon cancer, breast cancer, bladder cancer, skin papilloma, pancreatic carcinoma, lung cancer	Mo et al., 2007; Sun et al., 2007; Coppé et al., 2008; Vredeveld et al., 2012; Basu et al., 2018; Nacarelli et al., 2020; Wang et al., 2020
Raf	Up	Melanoma, thyroid cancer, colon carcinoma	Zhu et al., 1998; Chan et al., 2013
EGFR	Up	Glioblastoma, melanoma, esophageal squamous cell carcinoma, lung cancer, EBV-positive nasopharyngeal carcinoma	Hotta et al., 2007; Ohashi et al., 2010; Bian et al., 2012; Sun et al., 2014; Liu et al., 2017
PTEN	Down	Prostate cancer, glioblastoma, lung cancer, head and neck squamous carcinoma	Chen et al., 2005; Banito et al., 2009; Lee et al., 2011; Ren et al., 2014; Revandkar et al., 2016
Rheb	Up	Prostate cancer	Nardella et al., 2008
E2f3	Up	Lung cancer, melanoma, ovarian cancer	Nowicki et al., 2011; Noguchi et al., 2012; Weiner-Gorzel et al., 2015
Akt1	Up	Esophageal epithelial carcinoma, Breast cancer, Papillary thyroid carcinoma	Oyama et al., 2007; Wu et al., 2007; Hayes et al., 2016
Myc	Down	Lymphoma, osteosarcoma, liver carcinoma, lung carcinoma, pancreatic cancer	Soucek et al., 2008; Zinke et al., 2015; Takasugi et al., 2017; Zhang et al., 2019
β-catenin	Up	Medulloblastoma, glioma, lung cancer	Bikkavilli et al., 2015; Giménez-Bastida et al., 2019; Zhang et al., 2019
Rb	Down	Breast cancer, pancreas cancer, ovarian cancer, head and neck squamous cell carcinoma, gastric cancer	Ventura et al., 2007; Xing et al., 2012; Bikkavilli et al., 2015; Macha et al., 2015; Marino Gammazza et al., 2017; Martins et al., 2018
P53	Up/Down	Sarcoma, liver carcinoma, non-small cell lung cancer, breast cancer, ovarian cancer, colorectal cancer, cervical carcinoma	Chen et al., 2005; Xue et al., 2007; Nekulova et al., 2011; Macha et al., 2015; Marino Gammazza et al., 2017
P63	Up	Skin cancer, head and neck cancer, lung cancer, Barrett’s adenocarcinoma	Wu et al., 2020b
Vhl	Down	Renal cell carcinoma	Young et al., 2008
SKP2	Down	Glioma, head and neck squamous cell carcinoma, prostate cancer, mammary gland cancer, gastric cancer, pancreatic ductal adenocarcinoma	Pernicova et al., 2011; Grasso et al., 2014; Kim et al., 2014; Seo et al., 2019
RAC1	Up	Breast cancer, colorectal tumor	Henriques et al., 2015; Liu et al., 2015
MOS	Up	Osteosarcoma	Bartkova et al., 2006
MEK	Up	Colon cancer, pancreas cancer, gastric cancer	Ventura et al., 2007; Zinke et al., 2015; Wang et al., 2018
CDC6	Up	Pancreatic cancer	Lim and Townsend, 2020
Cyclin E	Up	Osteosarcoma	Bartkova et al., 2006
Hsp72	Down	Breast cancer, colon cancer, prostate cancer	Yaglom et al., 2007
TGF-β	Up	Non–small cell lung carcinoma	Katakura et al., 1999
Cdt1	Up	Non–small cell lung carcinoma, head and neck carcinoma, colon cancer	Liontos et al., 2007
DEC1	Up	Breast cancer, colon cancer	Qian et al., 2008

Subsequently, over-expression or ectopic expression of other oncogenes, including RAS, RAF, BRAF, MEK, ARF, MOS, E2F1, CDC6, and cyclin E has been shown to cause senescence (Lin et al., 1998; Zhu et al., 1998; Dimri et al., 2000; Michaloglou et al., 2005; Bartkova et al., 2006; Coppe et al., 2008). Tumor suppressors PTEN, NF1, VHL, and RB constitutively reduce pro-oncogenic signaling from PI3K, HIF1α, RAS, and E2F, respectively. Therefore, the inactivation of these tumor suppressors leads to the accumulation of cells expressing markers of senescence (Chen et al., 2005; Courtois-Cox et al., 2006; Young et al., 2008). Although a lot of studies have focused on the genes affecting senescence, the non-genetic regulation of senescence is attracting increasing attention. Senescence has been shown to be associated with profound changes in chromatin organization (Adams, 2009). One possible mechanism is that the disruption of heterochromatin by global chromatin relaxation may induce senescence (Roediger et al., 2010).

Concerning the molecular mechanisms responsible for induction of senescence following oncogene activation, several mechanisms have been proposed. It has been suggested that OIS is caused by accumulation of DNA damage and activation of the DDR. For example, one of the studies showed that the RAS protein may indirectly up-regulate the levels of mitochondrial ROS, which is a well-known DNA-damaging effect, while other studies reported that DNA damage is caused by oncogene-induced DNA replication stress (Lee et al., 1999; Bartkova et al., 2006; Di Micco et al., 2006). Previous studies have also shown that ROS may trigger senescence through phosphorylation of p53 by PRAK (p38-regulated/activated protein kinase) following activation of p38MAPK (Figure 2; Sun et al., 2007). Furthermore, senescence-associated heterochromatic foci (SAHFs) are formed in the process of OIS through a multi-step process and are thought to prevent transcription of E2F target genes involved in cell-cycle entry and proliferation (Narita et al., 2003). In addition, oncogenic events also promote senescence by inducing a global negative feedback response that potently suppresses the RAS/PI3K pathway (Courtois-Cox et al., 2006). Remarkably, these mechanisms are not necessarily mutually exclusive. Conversely, it is possible that multiple mechanisms cooperate to promote or maintain the senescence response (Courtois-Cox et al., 2008).

## Characteristics of Senescent Cells

Cellular senescence is an irreversible cell cycle arrest of previously replication-competent cells induced by stress, but the cells remain metabolically active. In addition to growth arrest, senescent cells are characterized by a set of features including morphological characteristics, expression of anti-proliferative molecules (e.g., p16INK4a), DNA-damage foci (e.g., TIF, DNA-SCARS), and SASP (e.g., IL-1, IL-6, and IL-8). Since there is no single characteristic that can robustly identify senescent cells, identification of senescent cells requires the combination of markers and features. Senescent cells have been observed in tumor masses. Senescent tumor cells usually exist in the marginal regions of the tumor, metastatic lymph nodes and lymphatic vessels, but not present in the center of tumor lesions (Kim et al., 2017).

Senescent cells are metabolically active and have an enlarged size, reflecting the continuation of macromolecule synthesis without cell division (Loaiza and Demaria, 2016). Furthermore, senescent cells exhibit flattened morphology with a prominent nucleus and increased cytoplasmic granularity (Cadenas et al., 2012; Campisi, 2013). Senescence-associated β-galactosidase is a manifestation of residual lysosomal activity under suboptimal pH, and it can be detected due to increased lysosomal content in senescent cells (Kurz et al., 2000). Senescence-associated β-galactosidase-positive cells accumulate with age in the skin of healthy individuals (Dimri et al., 1995). The discovery of high senescence-associated β-galactosidase activity in senescent cells provides a common marker for their detection in culture and in tissues (Dimri et al., 1995). It is notable that increased senescence-associated β-galactosidase activity is an outcome rather than a cause of senescence (Lee et al., 2006).

Gene expression profiles are profoundly affected during cellular senescence. p16INK4a, a selective inhibitor of cyclin D-dependent CDK4 and CDK6, is a commonly used marker for identifying senescent cells (Serrano et al., 1993). p16INK4a is encoded by CDKN2A gene, whose exons 2 and 3 also encode p14^ARF^ in human and p19^ARF^ in mouse (Quelle et al., 1995). The products of the CDKN2A locus can regulate the two central growth control pathways, RB and p53, playing a role in inducing cell cycle arrest (Pomerantz et al., 1998; Zhang et al., 1998; Chandler and Peters, 2013). p16INK4a is usually absent in healthy tissues of young animals, but is highly expressed in the senescent cells and tissues (Ohtani et al., 2004; Sharpless and Sherr, 2015). Therefore, p16INK4a could serve as a maker of cellular senescence. Moreover, another well-known change in gene expression in senescent cell is the activation of p53-p21 axis (Rufini et al., 2013).

As noted before, growing evidence indicates that senescence triggered by different stimuli is the result of protracted DNA damage. Dysfunctional telomeres-derived DNA damage foci is termed telomere dysfunction–induced foci (TIF), including γH2AX and 53BP1 foci localized in telomeres, which can be used to identify senescent cells in culture and tissues (Takai et al., 2003; Herbig et al., 2004, 2006). However, TIF can also be identified in pre-senescent cells and the damage foci in cells with premature senescence (Verdun et al., 2005; Beliveau et al., 2007; Nakamura et al., 2008; Wang et al., 2009; Passos et al., 2010). Senescent cells of multiple human cell types and mouse tissues also always harbor nuclear DNA segments with chromatin alterations reinforcing senescence (DNA-SCARS), which sustains DDR signaling and regulates multiple aspects of the senescent phenotype, including growth arrest and inflammatory cytokine secretion (Rodier et al., 2009, 2011; Gonzalez et al., 2016).

SAHFs are another heteromatin signature of senescent cells, which are associated with S-phase-promoting gene loci, such as E2F target genes (Narita et al., 2003; Sharpless and Sherr, 2015). DNA staining of normal cells shows completely uniform color outlines, while senescent cells usually display punctate patterns due to the formation of SAHF which may provide a chromatin buffer to prevent activation of proliferation-associated genes (Narita et al., 2003, 2006). The formation of SAHFs is due to the remodeling of chromatin and decreased sensitivity to nuclease digestion, and this process coincides with the recruitment of heterochromatin proteins and Rb to E2F-responsive promoters (Narita et al., 2003). It should be noted that SAHFs are dispensable for cellular senescence, and its existence depends on cell type and stimulation (Kosar et al., 2011).

Senescent cells, including but not limited to senescent fibroblasts, have an altered secretion pattern and secrete growth factors (e.g., HGF, GM-CSF, and TGFβ), chemokines (e.g., CXCL-1, CXCL -3, and CXCL-10), cytokines (e.g., IL-1, IL-6, and IL-8), and proteases, collectively known as SASP (Althubiti et al., 2014; Malaquin et al., 2016). The initiation of SASP is largely dependent on persistent DDR signal, phosphorylation of p38MAPK, and activation of transcription factors nuclear factor κB (NF-κB) (Rodier et al., 2009; Chien et al., 2011; Freund et al., 2011; Kang et al., 2015). In contrast, the inactivation of p53 in senescent cells causes an excessive increase in the secretion of several SASP factors (Coppe et al., 2008). Notably, cellular senescence induced by ectopic p16INK4a expression may not always harbor SASP (Coppe et al., 2011). SASP has effects on cell proliferation and angiogenesis, and may play a role in promoting aging, tumorigenesis and tumor metastasis (Krtolica et al., 2001; Zlotnik, 2004; Acosta et al., 2008).

## Senescence and Cancer

**FIGURE 3:**
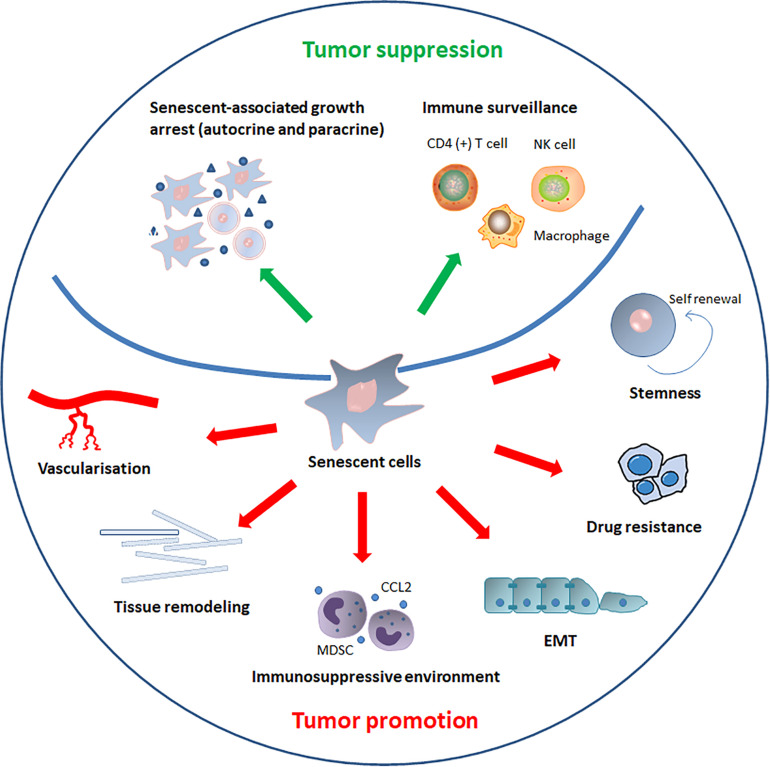
The role of senescence in cancer. SASP components induce or enhance the senescent-associated growth arrest in autocrine and paracrine manners, thereby inhibiting cancer progression. In addition to reinforcing senescence, SASP can also activate immune surveillance, which is orchestrated by specific immune responses mediated by antigen-specific CD4 (+) T cells. SASP also recruits natural killer (NK) cells and alters macrophage polarization to eliminate senescent tumor cells and suppress tumorigenesis. By contrast, senescent cells and SASP components can directly or indirectly promote tumor cells growth, invasion and metastasis by promoting tumor vascularization, maintaining stem-cell features, creating an immunosuppressive environment, remodeling tissue structure, inducing drug resistance, and stimulating epithelial-mesenchymal transition (EMT).

Cellular senescence is a well-defined process that plays a critical role in cancer (Perez-Mancera et al., 2014). Interferon-dependent induction of senescence-inducing cell cycle regulators are required to control cancer cells that escape from killing by immune checkpoint blocking or natural cancer immune response (Brenner and Schörg, 2020). Conventionally, senescence has been regarded as an irreversible mechanism of cell-cycle arrest that can protect against cancer (van Deursen, 2014). However, recent discoveries have led to different views regarding the role of senescence in the development of cancer. The role of senescence in cancer progression is summarized in the Figure 3.

### Cellular Senescence as a Tumor Suppressor

Genome instability is a major driving force of age-related diseases and cancer, posing a threat to human health and longevity. However, some stringent and complex cellular procedures maintain genomic integrity and reduce the risk for neoplastic transformation. Senescence is one of the obstacles to oncogenesis because senescent cells cannot respond to mitotic signals and cannot re-enter the cell cycle (Faggioli et al., 2018). In mice whose cells do not respond to senescence signals or whose genes encoding p53 or INK4a proteins are inactivated, cells fail to senesce in response to multiple stimuli; meanwhile, all the mice develop cancer at an early age (Ghebranious and Donehower, 1998). Furthermore, a positive correlation between the procreative life-span of mammary epithelial cells and cancer risk has been observed (Boulanger and Smith, 2001). Abrogation of OIS leads to aggressive cancer development (Kang et al., 2011; Mudbhary et al., 2014). When in a state of permanent cell cycle arrest, damaged or stressed cells cannot divide to form a tumor; thus, senescence represents a potent tumor suppressor mechanism (Campisi, 2001; Lowe et al., 2004).

The questions about the triggers of senescence during the early stages of malignant transformation are raised. The most common view is that OIS is an early protective barrier to prevent excessive proliferation of transformed cells, before telomeric abnormalities take effect (Collado and Serrano, 2010; Peeper, 2011). Other studies have shown that telomere dysfunction of cancer cell blocks apoptosis but limits cancer progression by inducing senescence (Cosme-Blanco et al., 2007; Feldser and Greider, 2007). However, it is unclear whether stress-induced senescence, replicative senescence, or both play a major role in inhibiting the proliferation of precancerous cells (Falandry et al., 2014). Previous studies have suggested that OIS is the first barrier to prevent excessive proliferation (Falandry et al., 2014). If this mechanism of senescence fails, the proliferation of precancerous cells will resume, leading to telomere dysfunction and growth crisis marked by failure to proliferate again (Zhu et al., 1999; Falandry et al., 2014). Nevertheless, active telomerase can avert this crisis and allow cell proliferation without requiring lengthening of telomeres, which increase the chance of malignant progression (Kolquist et al., 1998; Zhu et al., 1999).

Human melanoma that progressed rapidly during the blockade of immune checkpoint has the loss of senescence-inducing genes and amplification of senescence inhibitors (Brenner and Schörg, 2020). Suppression of cell senescence induces cisplatin resistance in ovarian cancer (Sun et al., 2020). The anti-tumor effect of senescence is mostly attributed to the lack of proliferative capacity of senescent cells (Perez-Mancera et al., 2014). Meanwhile, more and more evidences suggest that several SASP components induce or enhance senescent-associated growth arrest in autocrine and paracrine manners, produce a pro-inflammatory environment, and play a key role in propagating senescence and recruiting immune cells, thereby inhibiting cancer progression (Capece et al., 2018) (Lasry and Ben-Neriah, 2015). Of importance, several types of cytokines, such as IL-1, IL-6, and IL-8, play an important role in maintaining the SASP response of senescent cells themselves and the affected tissues (Acosta et al., 2008; Coppe et al., 2008). For example, inflammasome-mediated IL-1 is a key regulator of senescence that controls activation of the SASP program (Orjalo et al., 2009; Gross et al., 2012; Acosta et al., 2013). Blocking IL-1 or other inflammasome component caspase-1 prevents the induction of SASP during cell senescence (Gross et al., 2012). Therefore, IL-1 may maintain senescence and induce the inflammatory components of the SASP in an autocrine manner, resulting in tumor suppression.

Senescent cells actively communicate with neighboring cells and extracellular matrix through SASP, which has been reported to have anti-tumorigenic effects (Krtolica et al., 2001). When co-culture with senescent cells, normal cells displayed high DNA and oxidative damage, and activation of p16INK4a, p21cip1, and IL-8; paracrine senescence can be transmitted between different cell types (Acosta et al., 2013). Furthermore, in culture, mouse models and human of OIS, SASP can induce paracrine senescence in normal cells; multiple SASP components including transforming growth factor-β (TGF-β) family ligands, chemokine (C-C motif) ligand 2 (CCL2), chemokine (C-C motif) ligand 20 (CCL20) and vascular endothelial growth factor (VEGF), are identified to mediate paracrine senescence (Acosta et al., 2013). Thus, it is conceivable that, SASP could cause paracrine senescence and has an impact on tumor suppression and senescence during the early stage of tumorigenesis (Acosta et al., 2013; Perez-Mancera et al., 2014).

In addition to reinforcing senescence, SASP can also activate immune surveillance, which has been demonstrated in a mosaic mouse model for hepatocellular carcinoma (Xue et al., 2007; Kang et al., 2011). Induction of senescence by p53 activation in malignant hepatocytes contributes to tumor clearance through SASP-mediated differentiation and up-regulation of inflammatory cytokines (Xue et al., 2007). Moreover, this program only produces cell cycle arrest *in vitro*, and triggers an innate immune response against tumor cells *in vivo* (Xue et al., 2007). Pre-malignant senescent hepatocytes secrete chemicals and cytokines, and are cleared by liver-infiltrating immune cells, which is termed senescence surveillance and represents an important mechanism of the senescence anti-tumor barrier (Kang et al., 2011). This senescence surveillance is orchestrated by specific immune responses mediated by antigen-specific CD4 (+) T cells (Kang et al., 2011). On the other hand, SASP recruits natural killer (NK) cells and alters macrophage polarization to eliminate senescent tumor cells and suppress tumorigenesis (Iannello et al., 2013; Lujambio et al., 2013). Of note, p53 is of great significance in modifying the immune cells.

Overall, previous findings support the notion that senescence can be a barrier to tumor development. Firstly, as senescence is a state of permanent cell cycle arrest, damaged or stressed cells cannot divide to form a tumor. Secondly, several SASP components can induce or enhance the senescent-associated growth arrest in both autocrine and paracrine manners to help establish senescence-induced growth arrest. Thirdly, SASP can stimulate the immune system to target premalignant or malignant cells to suppress tumor progression. Recently, the bromo and extra terminal domain (BET) protein BRD4 has been reported to be required for the SASP, senescence-associated immune surveillance, and tumor-suppressive senescence program (Tasdemir et al., 2016).

### Cellular Senescence as a Tumor Promoting Factor

Age is an important independent risk factor for most human cancers, and the incidence of many types of cancer increases significantly with age. Although the link between aging and cancer is largely unknown, the cellular senescence may be one of the most important mechanisms, considering that senescent cells increase with age in mammalian tissues (Mo et al., 2007). There is growing evidence that the accumulation of senescent cells contributes to the progression of tumor (Wiley, 2020). Firstly, senescent fibroblasts promote premalignant and malignant epithelial cells to form tumors, partly in a SASP-dependent manner (Krtolica et al., 2001). Secondly, SASP components can directly or indirectly promote tumor cells growth, invasion and metastasis, and tumor vascularization (Davalos et al., 2010). Thirdly, senescence is associated with stem-cell-related properties and reprogramming of malignant cells (Milanovic et al., 2018). Recently, studies have found that senescent cells are tumor promoters, not tumor initiators, and that senescent cells stimulate skin cancer by up-regulating p38MAPK and MAPK/ERK signaling (Alimirah et al., 2020).

Senescent fibroblasts facilitate the growth of preneoplastic and neoplastic epithelial cells, but do not stimulate normal epithelial cells (Krtolica et al., 2001). The secretory phenotype of senescent fibroblasts accounts for at least 50% of growth stimulation (Krtolica et al., 2001). In mice, senescent fibroblasts strongly accelerate tumorigenesis (Krtolica et al., 2001). The ability of senescent fibroblasts to favor development has also been confirmed in a subsequent study showing that premalignant breast epithelial cells irreversibly lose differentiation characteristics, gain invasiveness and undergo malignant transformation when co-cultured with senescent human fibroblasts (Coppé et al., 2008). Senescent fibroblasts can impair epithelial morphological and functional differentiation in part by stimulating proliferation, migration and invasion of epithelial cells *via* collagen matrix, and it can also disrupt branching morphogenesis by increasing the secretion of MMP-3 (Coppé et al., 2008). Senescent carcinoma-associated fibroblasts promote pancreatic cancer invasion and metastasis partly through over-expression of IL-8 (Wang et al., 2017). Senescent fibroblasts secrete biologically active VEGF, a potent angiogenic factor that is effective in tumor vascularization and cancer progression (Nardella et al., 2008). Senescent fibroblasts augment the number of blood vessels and increase the size as well (Nardella et al., 2008). Mechanistically, senescence may synergize with hypoxia to induce VEGF production, but the levels of hypoxic-inducible (transcription) factor 1 α (HIF-1α) are very little increased (Nardella et al., 2008).

Different SASP components may have different biological activities, depending in part on the cell type and initiating events (Rao and Jackson, 2016). Although SASP can suppress tumor formation by blocking the cell division and promoting immune clearance of damaged cells, it also promotes cancer development through creating an immunosuppressive environment that contributes to tumor progression and relapse (Rao and Jackson, 2016; Li et al., 2020). A mouse model that mimics the aged skin microenvironment was developed to determine whether senescent stromal cells affected tumorigenesis (Ruhland et al., 2016). It was found that senescent stromal cells contribute to tumor promotion through driving localized increases of suppressive myeloid cells and creating an immuno-suppressed and tumor-permissive environment (Ruhland et al., 2016). The stromal-derived SASP component IL-6 could increase the number of myeloid-derived suppressor cells (MDSCs) and enhance the ability of MDSCs to inhibit anti-tumor T-cell responses (Ruhland et al., 2016). Moreover, SASP remodel tissue structure, disrupt local tissue integrity, and support tumor cell invasion and metastasis (Rodier and Campisi, 2011; Lecot et al., 2016). Senescent cells secrete a large number of proteases to degrade extracellular matrix, which makes the tissue structure more relaxed, thereby promoting the invasion of cancer cells (Lecot et al., 2016).

CCL2, also known as MCP-1, is an important factor secreted by senescent cells, and acts as a chemokine to recruit immune cells expressing receptor CCR2 (Acosta et al., 2008; Kang et al., 2011). Senescence-recruited CCR2 positive myeloid cells enhance hepatocellular carcinoma growth and worsen the prognosis of patients with hepatocellular carcinoma through NK cell inhibition (Eggert et al., 2016). The SASP components, VEGF, IL-8, I-309, and eotaxin, directly facilitate the proliferation and assembly of endothelial cells for neo-angiogenesis (Davalos et al., 2010). In addition to directly promote tumor vascularization, senescent cells can recruit macrophages and stimulate them to adopt the proangiogenic M2 phenotype, thus promoting tumor vascularization indirectly (Oyama et al., 2007).

The cytokines IL-6 and IL-8 are well-studied components of SASP that can stimulate inflammation, epithelial-to-mesenchymal transition (EMT) and invasiveness. Senescent cells may play a tumorigenic role as a source of IL-6 (Coppé et al., 2008). IL-6 cooperates with the transcription factor C/EBPβ to enhance the activation of the inflammatory network, including IL-8 (Kuilman et al., 2008). Senescent mesenchymal stem cells stimulate proliferation and migration of breast cancer cells *in vitro* and promote tumor progression in xenograft mice by activating IL-6/STAT-3 signaling pathway (Di et al., 2014; Zacarias-Fluck et al., 2015; Liu et al., 2017). Another study suggested that SASP induce EMT and invasiveness through a paracrine manner that relies mainly on IL-6 and IL-8 based on the observations that addition of IL-6 and IL-8 stimulates invasiveness while blocking them leads to a decrease in invasiveness (Coppe et al., 2008).

Senescent cells arise constantly in HER-2 positive breast cancer, accounting for about 5% of tumor cells (Korkaya et al., 2012). Blocking IL-6 impairs the growth of tumor, indicating the ability of IL-6 secreted by senescent cells in promoting cancer progression (Korkaya et al., 2012; Li et al., 2018). Furthermore, IL-6 may cooperate with HER2 in the process of tumor development (Korkaya et al., 2012; Tivari et al., 2018). *In vitro* dormancy model of MCF-7 breast cancer cells, bone marrow stroma secretory senescence (IL-6, IL-8, and TGFβ1) could reactivate dormant MCF-7 cells by promoting their phenotype to mesenchymal appearance, resulting in cellular proliferation and migration (Sun et al., 2018). The EMT genetic program is activated *via* reducing the expression of E-cadherin and increasing the expression of N-cadherin and SLUG before reactivation of dormant cells (Sun et al., 2018).

Cisplatin induces melanoma cell senescence and SASP *in vitro* and in melanoma xenograft mice (Chen et al., 2009). The cisplatin-induced senescent melanoma cells activate the ERK1/2-RSK1 pathway through SASP components (such as IL-8 and IL-1α) to promote the growth of non-senescent melanoma cells (Chen et al., 2009). Transplantation of non-senescent and senescent melanoma cells accelerates tumor growth compared to transplantation of non-senescent cells only to mice, whereas transplantation of senescent cells alone does not produce tumors (Chen et al., 2009).

It has been reported that functions of senescence and stem-cell appear to be regulated by overlapping signaling networks (Milanovic et al., 2018; Wang et al., 2020). The key senescence-related signaling molecules, such as p53, p16INK4a, and p21 also play an important role in the maintenance of stem-cell functions, which may have profound impact on tumor invasiveness (Medema, 2018; Milanovic et al., 2018; Wang et al., 2020). Further investigations indicated that upon entering cellular senescence, cancer cells of various tissue types acquire novel stemness-related properties (Yamakoshi et al., 2009; Milanovic et al., 2018). In lymphomas treated with the same dose of chemotherapy, previous senescent cells display a higher tumor-initiating potential than never senescent cells. Furthermore, when re-expose to chemotherapy drugs, previously senescent cells typically retain the ability to re-enter TIS (Milanovic et al., 2018). Notably, those results can also be observed even when the levels of SASP reduce drastically, indicating a cell-intrinsic mechanism of senescence-associated reprogramming (Milanovic et al., 2018). Wnt signaling is activated in TIS; this signaling plays a central role in stem-cell renewal in many tissues and is an essential driver of the enhanced tumor initiation capacity (Basu et al., 2018; Milanovic et al., 2018; Nacarelli et al., 2020). TIS contributes to chemo-resistance by inducing cancer stem-like cells (Ritschka et al., 2017).

In addition to maintaining stem-cell features of tumor cells with pre-existing self-renewal capabilities, cellular senescence can also promote the cell-autonomous reprogramming of non-stem cancer cells into cancer stem cells (Mosteiro et al., 2016; Milanovic et al., 2018). The transgenic expression of the four transcription factors Oct4, Sox2, Klf4, and c-Myc (OSKM) can induce two opposite cellular fates, cellular senescence and reprogramming (Banito et al., 2009; Mosteiro et al., 2018; Sai et al., 2020). IL-6 is a critical mediator of senescence-induced cellular reprogramming (Xu et al., 2018; Sai et al., 2020). Genetic locus INK4a is required for OSKM-induced senescence, IL-6 production, and reprogramming, whereas in the absence of p53, INK4a is not necessary (Xu et al., 2018).

Extracellular vesicles, heterogeneous populations of membrane vesicles, have emerged as new participants in intercellular communication; the exosome-like small extracellular vesicles have been demonstrated to be important mediators of the pro-tumorigenic function of senescent cells (Wu et al., 2007; Soucek et al., 2008; Takasugi et al., 2017; Han et al., 2020). In senescent cells, small extracellular vesicles sorting of EphA2 is increased due to increased phosphorylation caused by oxidative inactivation of PTP1B phosphatase (Han et al., 2020). EphA2 secreted from senescent cells binds to ephrin-A1, leading to cellular proliferation *via* EphA2/ephrin-A1 reverse signaling (Han et al., 2020). Furthermore, senescent stromal cells produce a large number of small extracellular vesicles, which change the expression profile of recipient cancer cells, thus enhancing the aggressiveness of cancer cells and promoting drug resistance in therapeutic settings (Ruscetti et al., 2020).

## Therapy-Induced Cellular Senescence

Therapy-induced senescence (TIS) appears as a result of pharmacological intervention that may end either in an advantageous outcome or an unwanted side effect. Senescence-induced therapies may be particularly effective when combined with chemotherapy and/or radiotherapy in the treatment of cancer. Therefore, TIS is one of the alternative strategies to improve the prognosis of patients, and low dose of TIS-induced drugs can achieve the purpose of inducing senescence, which means less off-target toxicity for patients (Ewald et al., 2010; Gonzalez et al., 2016). TIS is beneficial in cancer therapy in a certain extent, but it may bring risks over time. Increasing evidence has indicated that senescence induced by chemotherapy and radiotherapy is associated with treatment outcomes in various cancer types (Martins et al., 2018; Mongiardi et al., 2019). In addition, the significance of senescence induction in molecular targeted therapy and immunotherapy has been reported (Meng et al., 2012; Dorr et al., 2013; Wang and Gao, 2019; Wagner and Gil, 2020). Here, we reviewed the main literatures describing premature senescence as the primary mechanism of chemotherapy, radiotherapy, targeted therapy, or immunotherapy in preclinical and in clinical studies. Also, the related molecular mechanisms were summarized.

Many types of chemotherapeutic drugs can induce senescence of tumor cells *in vitro* and *in vivo*, which is considered to be a positive therapeutic outcome (Chang et al., 2002; Marino Gammazza et al., 2017). A previous study reported that lymphomas respond to cyclophosphamide through senescence induction; senescence-associated β-galactosidase was detected in lymphomas post-treatment and gradually accumulated, while it was not examined in untreated lymphomas (Marino Gammazza et al., 2017). Furthermore, the independent senescence marker PML was up-regulated in cyclophosphamide-treated lymphomas (Marino Gammazza et al., 2017). This cyclophosphamide-induced senescence was associated with outcomes of therapy and was highly dependent on the activity of p53- and p16INK4a, owing to the observations that deletion of the p53 or INK4a gene blocked premature senescence (Marino Gammazza et al., 2017). Doxorubicin, a drug commonly used in clinical chemotherapy, has been reported to induce replicative senescence in a human lung mucoepidermoid cell line (NCI-H292) (Song et al., 2019).

Cisplatin has been reported to induce cellular senescent-like growth arrest in nasopharyngeal carcinoma cell line, and senescence is the primary mechanism by which cisplatin induces wild-type TP53 head and neck squamous cell carcinoma cell responses (Gadhikar et al., 2013; Osman et al., 2015). Head and neck squamous cell carcinoma patients with TP53 mutations have a high risk of treatment failure after receiving cisplatin due to lack of senescence (Osman et al., 2015). Cisplatin induces G2 cell cycle arrest of cancer cell with TP53 mutations. Inhibition of wee-1 kinase (a tyrosine kinase involved in DNA damage–induced G2 cell cycle arrest) has been reported to enhance the efficacy of cisplatin in inhibiting p53 mutant tumor cells *in vivo* through eliminating G2 arrest and accumulating cells that carry unrepaired DNA damage during mitosis (Cerrito et al., 2018). These cells with unrepaired DNA damage cause abnormal cell division, leading to a senescence-like process without causing apoptosis (Cerrito et al., 2018). Furthermore, this senescence phenotype following MK-1775 addition depends on sustained ROS production rather than p21 expression (Cerrito et al., 2018).

Compared with traditional chemotherapy, metronomic chemotherapy cannot only reduce the side effects of drugs, but also improve the sensitivity and effectiveness of drugs. Metronomic combination of Vinorelbine and 5-Fluorouracil was able to inhibit triple-negative breast cancer cells, and significant increased cellular senescence was observed in the metronomic chemotherapy group compared with the standard therapy group (Taschner-Mandl et al., 2016). Metronomic chemotherapy can interfere with cell cycle regulation and DNA damage by inducing cancer cell senescence, mainly due to the activation of p53, the up-regulation of p21WAF/CIP1 and the inhibition of cyclin-dependent kinase, leading to permanent disruption of cell mitosis and cell cycle arrest (Demaria et al., 2017).

Contrary to the above results, some literatures have reported that chemotherapy-induced senescence plays a negative role in the treatment of cancer. Generally, the effects of genotoxic chemotherapies on proliferating cells are non-specific, which may also induce senescence of normal cells while inducing tumor cells senescence, leading to cancer recurrence and chemotherapy toxicities (Canino et al., 2012). Furthermore, the important role of senescence, especially SASP, in therapy resistance has also been reported (Yu et al., 2019; Chambers et al., 2021).

Acute ionizing radiation causes senescence and apoptosis of nasopharyngeal carcinoma cells (CNE2), but cancer cells exhibit premature senescence, EMT and radiation resistance after long-term ionizing radiation exposure (Yang et al., 2019). Cell division cycle 6 (CDC6), an essential regulator of DNA replication, is ectopically over-expressed in radioresistant CNE2 cells (Yang et al., 2019). In this study, CDC6 was demonstrated to cause radioresistance by regulating senescence and EMT (Yang et al., 2019). On the one hand, permanent cell growth arrest and senescence of cancer cell induced by ionizing radiation may be one of the mechanisms of tumor suppression. On the other hand, premature senescence of CNE2 after long-term ionizing radiation exposure may be associated with tumor cell invasion and migration.

An elective KDM5A (a histone demethylase) inhibitor has been reported to induce senescence and repress the proliferation of KDM5A-overexpressing breast cancer cell lines (Han, 2019). Mechanistically, this KDM5A inhibitor induces G1 cell cycle arrest of breast cancer cell and cellular senescence by up-regulation of p16 and p27 (Han, 2019). Another histone demethylase lysine specific demethylase 1 (LSD1) has also been demonstrated to induce senescence through down-regulation of hypoxia-inducible factor 1 (HIF-1) (Perez-Campo et al., 2014). Histone acetyltransferases lysine acetyltransferase 6A/B (KAT6A/B) are involved in cancer development. KAT6A plays an important role in maintaining the functions of normal hematopoietic stem cells and is a target for recurrent chromosomal translocations associated with acute myeloid leukemia (Baell et al., 2018; Ignacio et al., 2018). Also, the chromosomal translocations in KAT6B have been observed in several cancers (Sheikh et al., 2015). KAT6A inhibits cellular senescence by regulating inhibitors of the cdkn2a locus (Patten et al., 2019). KAT6B inhibits senescence by regulating INK4a-ARF locus encoding p16INK4a and p19ARF (Patten et al., 2019). WM-8014 and WM-1119, selective inhibitors of KAT6A and KAT6B, induce cell cycle arrest and senescence of lymphoma cells, which is not accompanied by DNA damage (Baell et al., 2018). Therefore, senescence induction is an important mechanism by which KAT6A/B inhibitors inhibit tumors. In addition, inhibiting the activity of many other proteins can also induce senescence, including receptor tyrosine kinase (RTK), casein kinase 2 (CK2), and Aurora A kinase (Huck et al., 2010; Francica et al., 2017; Yang et al., 2017).

Inhibitors of several classical cancer-related signaling pathways have also been reported to induce senescence, including inhibitors of VEGFRs, EGFR, MET, HER, AKT, and ERK (Alagkiozidis et al., 2017; Morelli et al., 2017). Axitinib, an inhibitor of VEGFRs, promotes senescence of glioblastoma cell line (U87MG) and human endothelial cells (Wang et al., 2011; Meng et al., 2012). Studies have reported that gefitinib (an inhibitor of EGFR tyrosine kinase) and cetuximab (monoclonal antibody anti EGFR) exert their antitumor effects through induction of cellular senescence *in vivo* and *in vitro* in non-small cell lung cancer, and this senescence program is independent from p53 and/or p16 function (Hotta et al., 2007; Francica et al., 2016). In pancreas cancer, the combination of MEK and CDK4/6 inhibitors can induce senescence and SASP (include pro-angiogenic factors), thereby enhancing drug delivery and efficacy of cytotoxic chemotherapy (Martins et al., 2018). The MET targeting-induced senescence phenotype has been shown to be associated with the inhibition of MAPK signaling pathway and the subsequent down-regulation of forkhead box protein M1 (FOXM1) transcription factor (De Martino and Tkach, 2020). Blocking of Stat3 oncogene addiction can induce cellular senescence, trigger antitumor immune responses and improve the effectiveness of immune checkpoint inhibitors (Coutu et al., 2011). Another example is that fibroblast growth factor receptors (FGFRs) signaling *via* PI3K/AKT pathway could inhibit senescence program by promoting the activation of murine double minute 2 (MDM2, a negative regulator of p53), and inhibition of FGFRs induces senescence (Alimonti et al., 2010).

The most important mechanism of TIS is to increase the amount or activity of tumor suppressors, mainly including p53, p21, p16, p27, and PTEN (Chen et al., 2005; Lee et al., 2010; Storer et al., 2013; Kalathur et al., 2015; Harajly et al., 2016; Park and Jeong, 2019). In addition, other genes such as Skp2 can also serve as backups to trigger TIS in the absence of p53 activity (Childs et al., 2015). The modulation of the activity of histone lysine acetyltransferases, histone demethylase, and several protein kinases involved in senescence are able to induce cell senescence. Certainty, modulation of these proteins always links to the regulation of the activity of tumor suppressors. Although senescence of tumor cells is considered to be a positive therapeutic outcome, the senescence of normal cells and related SASP are associated with tumor recurrence, drug resistance and occurrence of side effects. Therefore, direct killing of senescent cells by means of apoptosis or non-apoptosis, and blocking the effects of SASP, can greatly minimize the off-target effects of senescence and reduce the side effects (Laberge et al., 2015).

## Conclusion

Cellular senescence provides a significant benefit to the host by inducing irreversible cell cycle arrest and eliciting potent immune-mediated incipient tumor cell clearance, which is characterized by reduced incidence of cancer and halted tumor development. Senescence provides an alternative strategy to overcome the limitations of traditional cancer treatment because low dose of drugs can achieve the purpose of inducing senescence. However, senescent cells and SASP components can directly or indirectly promote tumor cells growth, invasion and metastasis, and tumor vascularization. The senescence phenotype is complicated, and the production rate and clearance rate of senescent cell may be the influencing factors of the effects of senescence on tumor progression. One of the possibilities is that senescent cells are only beneficial when they are transient, and the accumulation of senescent cells and SASP cause increased susceptibility to tumorigenesis.

The in-deep understanding and utilization of senescence in cancer therapy has gained increasing attention and has become an important research field. Previous findings have indicated that TIS is a positive outcome of therapy, since senescence is a state of growth arrest reflecting the loss of reproductive potential. In order to overcome the negative effects of TIS in cancer treatment, the concept of combining senescence-inducing therapies and removal of senescent cells, both normal and tumor derived, or manipulating the paracrine effects of SASP is proposed (Collado et al., 2005; Pribluda et al., 2013; Widemann and Italiano, 2018). However, before clinical application, we must balance the validity and potential risks, and determine the overall advantages of this treatment concept.

There are still issues needed to be addressed, although a lot of studies have focused on senescence. It is very important to determine under what circumstances senescent cells are beneficial or harmful to cancer treatment. Manipulating SASP is also essential, which may beneficial to anti-tumor.

## Author Contributions

JY and ML: data curation, formal analysis, and writing—original draft. DH: data curation and formal analysis. MZ: conceptualization and supervision. XZ: conceptualization, supervision, validation, and writing—review editing. All authors read and approved the final manuscript.

## Conflict of Interest

The authors declare that the research was conducted in the absence of any commercial or financial relationships that could be construed as a potential conflict of interest.

## Publisher’s Note

All claims expressed in this article are solely those of the authors and do not necessarily represent those of their affiliated organizations, or those of the publisher, the editors and the reviewers. Any product that may be evaluated in this article, or claim that may be made by its manufacturer, is not guaranteed or endorsed by the publisher.

## References

[B1] Acosta J. C. Banito A. Wuestefeld T. Georgilis A. Janich P. Morton J. P. (2013). A complex secretory program orchestrated by the inflammasome controls paracrine senescence. *Nat. Cell Biol.* 15 978–990. 10.1038/ncb2784 23770676 PMC3732483

[B2] Acosta J. C. O’Loghlen A. Banito A. Guijarro M. V. Augert A. Raguz S. (2008). Chemokine signaling via the CXCR2 receptor reinforces senescence. *Cell* 133 1006–1018. 10.1016/j.cell.2008.03.038 18555777

[B3] Adams P. D. (2009). Healing and hurting: molecular mechanisms, functions, and pathologies of cellular senescence. *Mol. Cell* 36 2–14. 10.1016/j.molcel.2009.09.021 19818705

[B4] Alagkiozidis I. Gorelick C. Shah T. Chen Y. A. Gupta V. Stefanov D. (2017). Synergy between paclitaxel and anti-cancer peptide PNC-27 in the treatment of ovarian cancer. *Ann. Clin. Lab. Sci.* 47 271–281. 28667027

[B5] Alimirah F. Pulido T. Valdovinos A. Alptekin S. (2020). Cellular senescence promotes skin carcinogenesis through p38MAPK and p44/42MAPK Signaling. *Cancer Res.* 80 3606–3619. 10.1158/0008-5472.can-20-0108 32641409 PMC7484313

[B6] Alimonti A. Nardella C. Chen Z. Clohessy J. G. Carracedo A. Trotman L. C. (2010). A novel type of cellular senescence that can be enhanced in mouse models and human tumor xenografts to suppress prostate tumorigenesis. *J. Clin. Invest.* 120 681–693. 10.1172/jci40535 20197621 PMC2827955

[B7] Allsopp R. C. Chang E. Kashefi-Aazam M. Rogaev E. I. Piatyszek M. A. Shay J. W. (1995). Telomere shortening is associated with cell division *in vitro* and *in vivo*. *Exp. Cell Res.* 220 194–200. 10.1006/excr.1995.1306 7664836

[B8] Althubiti M. Lezina L. Carrera S. Jukes-Jones R. Giblett S. M. Antonov A. (2014). Characterization of novel markers of senescence and their prognostic potential in cancer. *Cell Death Dis.* 5:e1528. 10.1038/cddis.2014.489 25412306 PMC4260747

[B9] Baell J. B. Leaver D. J. Hermans S. J. Kelly G. L. Brennan M. S. Downer N. L. (2018). Inhibitors of histone acetyltransferases KAT6A/B induce senescence and arrest tumour growth. *Nature* 560 253–257. 30069049 10.1038/s41586-018-0387-5

[B10] Banito A. Rashid S. T. Acosta J. C. Li S. Pereira C. F. Geti I. (2009). Senescence impairs successful reprogramming to pluripotent stem cells. *Genes Dev.* 23 2134–2139. 10.1101/gad.1811609 19696146 PMC2751980

[B11] Barascu A. Le Chalony C. Pennarun G. Genet D. Imam N. Lopez B. (2012). Oxidative stress induces an ATM-independent senescence pathway through p38 MAPK-mediated lamin B1 accumulation. *EMBO J.* 31 1080–1094. 10.1038/emboj.2011.492 22246186 PMC3297999

[B12] Barnoud T. Schmidt M. L. Donninger H. Clark G. J. (2017). The role of the NORE1A tumor suppressor in Oncogene-Induced Senescence. *Cancer Lett.* 400 30–36. 10.1016/j.canlet.2017.04.030 28455242 PMC5502528

[B13] Bartkova J. Rezaei N. Liontos M. Karakaidos P. Kletsas D. Issaeva N. (2006). Oncogene-induced senescence is part of the tumorigenesis barrier imposed by DNA damage checkpoints. *Nature* 444 633–637. 10.1038/nature05268 17136093

[B14] Basu S. K. Lee S. Salotti J. Basu S. Sakchaisri K. Xiao Z. (2018). Oncogenic RAS-induced perinuclear signaling complexes requiring KSR1 regulate signal transmission to downstream targets. *Cancer Res.* 78 891–908. 10.1158/0008-5472.can-17-2353 29259016 PMC5815900

[B15] Beausejour C. M. Krtolica A. Galimi F. Narita M. Lowe S. W. Yaswen P. (2003). Reversal of human cellular senescence: roles of the p53 and p16 pathways. *EMBO J.* 22 4212–4222. 10.1093/emboj/cdg417 12912919 PMC175806

[B16] Beliveau A. Bassett E. Lo A. T. Garbe J. Rubio M. A. Bissell M. J. (2007). p53-dependent integration of telomere and growth factor deprivation signals. *Proc. Natl. Acad. Sci. U.S.A.* 104 4431–4436. 10.1073/pnas.0700260104 17360541 PMC1838618

[B17] Ben-Porath I. Weinberg R. A. (2005). The signals and pathways activating cellular senescence. *Int. J. Biochem. Cell Biol.* 37 961–976. 10.1016/j.biocel.2004.10.013 15743671

[B18] Bian Y. Hall B. Sun Z. J. Molinolo A. Chen W. Gutkind J. S. (2012). Loss of TGF-beta signaling and PTEN promotes head and neck squamous cell carcinoma through cellular senescence evasion and cancer-related inflammation. *Oncogene* 31 3322–3332. 10.1038/onc.2011.494 22037217 PMC3270146

[B19] Bikkavilli R. K. Avasarala S. Van Scoyk M. Arcaroli J. Brzezinski C. Zhang W. (2015). Wnt7a is a novel inducer of beta-catenin-independent tumor-suppressive cellular senescence in lung cancer. *Oncogene* 34 5317–5328. 10.1038/onc.2015.2 25728679 PMC4558401

[B20] Bodnar A. G. Ouellette M. Frolkis M. Holt S. E. Chiu C. P. Morin G. B. (1998). Extension of life-span by introduction of telomerase into normal human cells. *Science (New York, N.Y.)* 279 349–352. 10.1126/science.279.5349.349 9454332

[B21] Boulanger C. A. Smith G. H. (2001). Reducing mammary cancer risk through premature stem cell senescence. *Oncogene* 20 2264–2272. 10.1038/sj.onc.1204312 11402321

[B22] Braig M. Lee S. Loddenkemper C. Rudolph C. Peters A. H. Schlegelberger B. (2005). Oncogene-induced senescence as an initial barrier in lymphoma development. *Nature* 436 660–665. 10.1038/nature03841 16079837

[B23] Brenner E. Schörg B. F. (2020). Cancer immune control needs senescence induction by interferon-dependent cell cycle regulator pathways in tumours. *Nat. Commun.* 11:1335. 10.1038/s41467-020-14987-6 PMC7067802 32165639

[B24] Cadenas C. Vosbeck S. Hein E. M. Hellwig B. Langer A. Hayen H. (2012). Glycerophospholipid profile in oncogene-induced senescence. *Biochim. Biophys. Acta* 1821 1256–1268. 22178194 10.1016/j.bbalip.2011.11.008

[B25] Campisi J. (2001). Cellular senescence as a tumor-suppressor mechanism. *Trends Cell Biol.* 11 S27–S31. 11684439 10.1016/s0962-8924(01)02151-1

[B26] Campisi J. (2013). Aging, cellular senescence, and cancer. *Annu. Rev. Physiol.* 75 685–705. 23140366 10.1146/annurev-physiol-030212-183653 PMC4166529

[B27] Campisi J. d’Adda di Fagagna F. (2007). Cellular senescence: when bad things happen to good cells. *Nat. Rev. Mol. Cell Biol.* 8 729–740. 10.1038/nrm2233 17667954

[B28] Canino C. Mori F. Cambria A. Diamantini A. Germoni S. Alessandrini G. (2012). SASP mediates chemoresistance and tumor-initiating-activity of mesothelioma cells. *Oncogene* 31 3148–3163. 10.1038/onc.2011.485 22020330

[B29] Capece D. Verzella D. Tessitore A. Alesse E. Capalbo C. Zazzeroni F. (2018). Cancer secretome and inflammation: The bright and the dark sides of NF-kappaB. *Sem. Cell Dev. Biol.* 78 51–61. 10.1016/j.semcdb.2017.08.004 28779979

[B30] Cerrito M. G. Pelizzoni D. Bonomo S. M. Digiacomo N. Scagliotti A. Bugarin C. (2018). Metronomic combination of Vinorelbine and 5-Fluorouracil inhibit triple-negative breast cancer cells results from the proof of- concept VICTOR-0 study. *Oncotarget* 9 27448–27459. 10.18632/oncotarget.25422 29937997 PMC6007943

[B31] Chambers C. R. Ritchie S. Pereira B. A. Timpson P. (2021). Overcoming the senescence-associated secretory phenotype (SASP): a complex mechanism of resistance in the treatment of cancer. *Mol. Oncol.* 10.1002/1878-0261.13042 Epub ahead of print, 34137158 PMC8637570

[B32] Chan K. C. Ting C. M. Chan P. S. Lo M. C. Lo K. W. Curry J. E. (2013). A novel Hsp90 inhibitor AT13387 induces senescence in EBV-positive nasopharyngeal carcinoma cells and suppresses tumor formation. *Mol. Cancer* 12:128. 10.1186/1476-4598-12-128 24156782 PMC3834878

[B33] Chandler H. Peters G. (2013). Stressing the cell cycle in senescence and aging. *Curr. Opin. Cell Biol.* 25 765–771. 10.1016/j.ceb.2013.07.005 23916530

[B34] Chang B. D. Swift M. E. Shen M. Fang J. Broude E. V. Roninson I. B. (2002). Molecular determinants of terminal growth arrest induced in tumor cells by a chemotherapeutic agent. *Proc. Natl. Acad. Sci. U.S.A.* 99 389–394. 10.1073/pnas.012602599 11752408 PMC117570

[B35] Chen X. Mitsutake N. LaPerle K. Akeno N. Zanzonico P. Longo V. A. (2009). Endogenous expression of Hras(G12V) induces developmental defects and neoplasms with copy number imbalances of the oncogene. *Proc. Natl. Acad. Sci. U.S.A.* 106 7979–7984. 10.1073/pnas.0900343106 19416908 PMC2674938

[B36] Chen Z. Trotman L. C. Shaffer D. Lin H. K. Dotan Z. A. Niki M. (2005). Crucial role of p53-dependent cellular senescence in suppression of Pten-deficient tumorigenesis. *Nature* 436 725–730. 10.1038/nature03918 16079851 PMC1939938

[B37] Chien Y. Scuoppo C. Wang X. Fang X. Balgley B. Bolden J. E. (2011). Control of the senescence-associated secretory phenotype by NF-kappaB promotes senescence and enhances chemosensitivity. *Genes Dev.* 25 2125–2136. 10.1101/gad.17276711 21979375 PMC3205583

[B38] Childs B. G. Durik M. Baker D. J. van Deursen J. M. (2015). Cellular senescence in aging and age-related disease: from mechanisms to therapy. *Nat. Med.* 21 1424–1435. 10.1038/nm.4000 26646499 PMC4748967

[B40] Collado M. Gil J. Efeyan A. Guerra C. Schuhmacher A. J. Barradas M. (2005). Tumour biology: senescence in premalignant tumours. *Nature* 436:642. 10.1038/436642a 16079833

[B41] Collado M. Serrano M. (2010). Senescence in tumours: evidence from mice and humans. *Nat. Rev. Cancer* 10 51–57. 10.1038/nrc2772 20029423 PMC3672965

[B42] Coppé J. P. Patil C. K. Rodier F. Sun Y. Muñoz D. P. Goldstein J. (2008). Senescence-associated secretory phenotypes reveal cell-nonautonomous functions of oncogenic RAS and the p53 tumor suppressor. *PLoS Biol.* 6:2853–2868. 10.1371/journal.pbio.0060301 19053174 PMC2592359

[B43] Coppe J. P. Patil C. K. Rodier F. Sun Y. Munoz D. P. Goldstein J. (2008). Senescence-associated secretory phenotypes reveal cell-nonautonomous functions of oncogenic RAS and the p53 tumor suppressor. *PLoS Biol.* 6:2853–2868. 19053174 10.1371/journal.pbio.0060301 PMC2592359

[B44] Coppe J. P. Rodier F. Patil C. K. Freund A. Desprez P. Y. Campisi J. (2011). Tumor suppressor and aging biomarker p16(INK4a) induces cellular senescence without the associated inflammatory secretory phenotype. *J. Biol. Chem.* 286 36396–36403. 10.1074/jbc.m111.257071 21880712 PMC3196093

[B45] Cosme-Blanco W. Shen M. F. Lazar A. J. Pathak S. Lozano G. Multani A. S. (2007). Telomere dysfunction suppresses spontaneous tumorigenesis in vivo by initiating p53-dependent cellular senescence. *EMBO Rep.* 8 497–503. 10.1038/sj.embor.7400937 17396137 PMC1866197

[B46] Courtois-Cox S. Genther Williams S. M. Reczek E. E. Johnson B. W. McGillicuddy L. T. Johannessen C. M. (2006). A negative feedback signaling network underlies oncogene-induced senescence. *Cancer Cell* 10 459–472. 10.1016/j.ccr.2006.10.003 17157787 PMC2692661

[B47] Courtois-Cox S. Jones S. L. Cichowski K. (2008). Many roads lead to oncogene-induced senescence. *Oncogene* 27 2801–2809. 10.1038/sj.onc.1210950 18193093

[B48] Coutu D. L. Francois M. Galipeau J. (2011). Inhibition of cellular senescence by developmentally regulated FGF receptors in mesenchymal stem cells. *Blood* 117 6801–6812. 10.1182/blood-2010-12-321539 21527526

[B49] d’Adda di Fagagna F. (2008). Living on a break: cellular senescence as a DNA-damage response. *Nat. Rev. Cancer* 8 512–522. 10.1038/nrc2440 18574463

[B50] d’Adda di Fagagna F. Reaper P. M. Clay-Farrace L. Fiegler H. Carr P. Von Zglinicki T. (2003). A DNA damage checkpoint response in telomere-initiated senescence. *Nature* 426 194–198. 10.1038/nature02118 14608368

[B51] Davalos A. R. Coppe J. P. Campisi J. Desprez P. Y. (2010). Senescent cells as a source of inflammatory factors for tumor progression. *Cancer Metastasis Rev.* 29 273–283. 10.1007/s10555-010-9220-9 20390322 PMC2865636

[B52] De Martino M. Tkach M. (2020). Blockade of Stat3 oncogene addiction induces cellular senescence and reveals a cell-nonautonomous activity suitable for cancer immunotherapy. *Oncoimmunology* 9:1715767. 10.1080/2162402x.2020.1715767 32064174 PMC6996562

[B53] Demaria M. O’Leary M. N. Chang J. Shao L. Liu S. Alimirah F. (2017). Cellular senescence promotes adverse effects of chemotherapy and cancer relapse. *Cancer Discov.* 7 165–176. 10.1158/2159-8290.cd-16-0241 27979832 PMC5296251

[B54] Denchi E. L. de Lange T. (2007). Protection of telomeres through independent control of ATM and ATR by TRF2 and POT1. *Nature* 448 1068–1071. 10.1038/nature06065 17687332

[B55] Di G. H. Liu Y. Lu Y. Liu J. Wu C. Duan H. F. (2014). IL-6 secreted from senescent mesenchymal stem cells promotes proliferation and migration of breast cancer cells. *PLoS One* 9:e113572. 10.1371/journal.pone.0113572 25419563 PMC4242635

[B56] Di Leonardo A. Linke S. P. Clarkin K. Wahl G. M. (1994). DNA damage triggers a prolonged p53-dependent G1 arrest and long-term induction of Cip1 in normal human fibroblasts. *Genes Dev.* 8 2540–2551. 10.1101/gad.8.21.2540 7958916

[B57] Di Micco R. Fumagalli M. Cicalese A. Piccinin S. Gasparini P. Luise C. (2006). Oncogene-induced senescence is a DNA damage response triggered by DNA hyper-replication. *Nature* 444 638–642. 10.1038/nature05327 17136094

[B58] Dimri G. P. Itahana K. Acosta M. Campisi J. (2000). Regulation of a senescence checkpoint response by the E2F1 transcription factor and p14(ARF) tumor suppressor. *Mol. Cell. Biol.* 20 273–285. 10.1128/mcb.20.1.273-285.2000 10594030 PMC85083

[B59] Dimri G. P. Lee X. Basile G. Acosta M. Scott G. Roskelley C. (1995). A biomarker that identifies senescent human cells in culture and in aging skin in vivo. *Proc. Natl. Acad. Sci. U.S.A.* 92 9363–9367. 10.1073/pnas.92.20.9363 7568133 PMC40985

[B60] Dorr J. R. Yu Y. Milanovic M. Beuster G. Zasada C. Dabritz J. H. (2013). Synthetic lethal metabolic targeting of cellular senescence in cancer therapy. *Nature* 501 421–425. 10.1038/nature12437 23945590

[B61] Dou Z. Berger S. L. (2018). Senescence elicits stemness: a surprising mechanism for cancer relapse. *Cell Metab.* 27 710–711. 10.1016/j.cmet.2018.03.009 29617638 PMC7205594

[B62] Eggert T. Wolter K. Ji J. Ma C. Yevsa T. Klotz S. (2016). Distinct functions of senescence-associated immune responses in liver tumor surveillance and tumor progression. *Cancer Cell* 30 533–547. 10.1016/j.ccell.2016.09.003 27728804 PMC7789819

[B63] Ewald J. A. Desotelle J. A. Wilding G. Jarrard D. F. (2010). Therapy-induced senescence in cancer. *J. Proteome Res.* 102 1536–1546. 10.1093/jnci/djq364 PMC2957429 20858887

[B64] Faggioli F. Palagano E. Di Tommaso L. Donadon M. Marrella V. Recordati C. (2018). B lymphocytes limit senescence-driven fibrosis resolution and favor hepatocarcinogenesis in mouse liver injury. *Hepatology (Baltimore, Md.)* 67 1970–1985. 10.1002/hep.29636 29105104

[B65] Falandry C. Bonnefoy M. Freyer G. Gilson E. (2014). Biology of cancer and aging: a complex association with cellular senescence. *J. Clin. Oncol. J. Am. Soc. Clin. Oncol.* 32 2604–2610. 10.1200/jco.2014.55.1432 25071126

[B66] Feldser D. M. Greider C. W. (2007). Short telomeres limit tumor progression in vivo by inducing senescence. *Cancer Cell* 11 461–469. 10.1016/j.ccr.2007.02.026 17433785 PMC1945093

[B67] Francica P. Aebersold D. M. Medova M. (2017). Senescence as biologic endpoint following pharmacological targeting of receptor tyrosine kinases in cancer. *Biochem. Pharmacol.* 126 1–12. 10.1016/j.bcp.2016.08.022 27574725

[B68] Francica P. Nisa L. Aebersold D. M. Langer R. Bladt F. Blaukat A. (2016). Depletion of FOXM1 via MET targeting underlies establishment of a DNA damage-induced senescence program in gastric cancer. *Clin. Cancer Res. J. Am. Assoc. Cancer Res.* 22 5322–5336. 10.1158/1078-0432.ccr-15-2987 27185371

[B69] Franza B. R. Jr. Maruyama K. Garrels J. I. Ruley H. E. (1986). *In vitro* establishment is not a sufficient prerequisite for transformation by activated ras oncogenes. *Cell* 44 409–418. 10.1016/0092-8674(86)90462-9 3510745

[B70] Freund A. Patil C. K. Campisi J. (2011). p38MAPK is a novel DNA damage response-independent regulator of the senescence-associated secretory phenotype. *EMBO J.* 30 1536–1548. 10.1038/emboj.2011.69 21399611 PMC3102277

[B71] Gadhikar M. A. Sciuto M. R. Alves M. V. Pickering C. R. Osman A. A. Neskey D. M. (2013). Chk1/2 inhibition overcomes the cisplatin resistance of head and neck cancer cells secondary to the loss of functional p53. *Mol. Cancer Therap.* 12 1860–1873. 10.1158/1535-7163.mct-13-0157 23839309 PMC3955083

[B72] Ghebranious N. Donehower L. A. (1998). Mouse models in tumor suppression. *Oncogene* 17 3385–3400. 10.1038/sj.onc.1202573 9917000

[B73] Giménez-Bastida J. A. Ávila-Gálvez M. Espín J. C. González-Sarrías A. (2019). Conjugated physiological resveratrol metabolites induce senescence in breast cancer cells: role of p53/p21 and p16/Rb Pathways, and ABC Transporters. *Mol. Nutr. Food Res.* 63:e1900629. 10.1002/mnfr.201900629 31441212

[B74] Gonzalez L. C. Ghadaouia S. Martinez A. Rodier F. (2016). Premature aging/senescence in cancer cells facing therapy: good or bad? *Biogerontology* 17 71–87. 10.1007/s10522-015-9593-9 26330289

[B75] Grasso D. Garcia M. N. Hamidi T. Cano C. Calvo E. Lomberk G. (2014). Genetic inactivation of the pancreatitis-inducible gene Nupr1 impairs PanIN formation by modulating Kras(G12D)-induced senescence. *Cell Death Differ.* 21 1633–1641. 10.1038/cdd.2014.74 24902898 PMC4158688

[B76] Gross O. Yazdi A. S. Thomas C. J. Masin M. Heinz L. X. Guarda G. (2012). Inflammasome activators induce interleukin-1alpha secretion via distinct pathways with differential requirement for the protease function of caspase-1. *Immunity* 36 388–400. 10.1016/j.immuni.2012.01.018 22444631

[B77] Han C. Y. (2019). p53 Promotes chemoresponsiveness by regulating hexokinase II gene transcription and metabolic reprogramming in epithelial ovarian cancer. *Mol. Carcinog.* 58 2161–2174. 10.1002/mc.23106 31486135

[B78] Han L. Long Q. Li S. Xu Q. Zhang B. Dou X. (2020). Senescent stromal cells promote cancer resistance through SIRT1 loss-potentiated overproduction of small extracellular vesicles. *Cancer Res.* 80 3383–3398. 10.1158/0008-5472.can-20-0506 32366480 PMC7611217

[B79] Harajly M. Zalzali H. Nawaz Z. Ghayad S. E. Ghamloush F. Basma H. (2016). p53 restoration in induction and maintenance of senescence: differential effects in premalignant and malignant tumor cells. *Mol. Cell. Biol.* 36 438–451. 10.1128/mcb.00747-15 26598601 PMC4719431

[B80] Harley C. B. Futcher A. B. Greider C. W. (1990). Telomeres shorten during ageing of human fibroblasts. *Nature* 345 458–460. 10.1038/345458a0 2342578

[B81] Hayes T. K. Neel N. F. Hu C. Gautam P. Chenard M. Long B. (2016). Long-Term ERK Inhibition in KRAS-mutant pancreatic cancer is associated with MYC degradation and senescence-like growth suppression. *Cancer Cell* 29 75–89. 10.1016/j.ccell.2015.11.011 26725216 PMC4816652

[B82] Hayflick L. (1965). The limited *in vitro* lifetime of human diploid cell strains. *Exp. Cell Res.* 37 614–636. 10.1016/0014-4827(65)90211-9 14315085

[B245] Hayflick L. Moorhead P. S. (1961). The serial cultivation of human diploid cell strains. *Exp. Cell Res.* 25 585–621. 13905658 10.1016/0014-4827(61)90192-6

[B83] Henriques A. F. Barros P. Moyer M. P. Matos P. Jordan P. (2015). Expression of tumor-related Rac1b antagonizes B-Raf-induced senescence in colorectal cells. *Cancer Lett.* 369 368–375. 10.1016/j.canlet.2015.08.027 26341689

[B84] Herbig U. Ferreira M. Condel L. Carey D. Sedivy J. M. (2006). Cellular senescence in aging primates. *Science (New York, N.Y.)* 311 1257. 10.1126/science.1122446 16456035

[B85] Herbig U. Jobling W. A. Chen B. P. Chen D. J. Sedivy J. M. (2004). Telomere shortening triggers senescence of human cells through a pathway involving ATM, p53, and p21(CIP1), but not p16(INK4a). *Mol. Cell* 14 501–513. 10.1016/s1097-2765(04)00256-4 15149599

[B86] Hotta K. Tabata M. Kiura K. Kozuki T. Hisamoto A. Katayama H. (2007). Gefitinib induces premature senescence in non-small cell lung cancer cells with or without EGFR gene mutation. *Oncol. Rep.* 17 313–317. 17203166

[B87] Huck J. J. Zhang M. McDonald A. Bowman D. Hoar K. M. Stringer B. (2010). MLN8054, an inhibitor of Aurora A kinase, induces senescence in human tumor cells both *in vitro* and *in vivo*. *Mol. Cancer Res. MCR* 8 373–384. 10.1158/1541-7786.mcr-09-0300 20197380

[B88] Iannello A. Thompson T. W. Ardolino M. Lowe S. W. Raulet D. H. (2013). p53-dependent chemokine production by senescent tumor cells supports NKG2D-dependent tumor elimination by natural killer cells. *J. Exp. Med.* 210 2057–2069. 10.1084/jem.20130783 24043758 PMC3782044

[B89] Ignacio R. M. C. Dong Y. L. Kabir S. M. Choi H. Lee E. S. Wilson A. J. (2018). CXCR2 is a negative regulator of p21 in p53-dependent and independent manner via Akt-mediated Mdm2 in ovarian cancer. *J. Gynecol. Oncol.* 9 9751–9765. 10.18632/oncotarget.24231 29515768 PMC5839399

[B90] Kalathur M. Toso A. Chen J. Revandkar A. Danzer-Baltzer C. Guccini I. (2015). A chemogenomic screening identifies CK2 as a target for pro-senescence therapy in PTEN-deficient tumours. *Nat. Commun.* 6:7227. 10.1038/ncomms8227 26085373

[B91] Kang C. Xu Q. Martin T. D. Li M. Z. Demaria M. Aron L. (2015). The DNA damage response induces inflammation and senescence by inhibiting autophagy of GATA4. *Science (New York, N.Y.)* 349 aaa5612. 10.1126/science.aaa5612 26404840 PMC4942138

[B92] Kang T. W. Yevsa T. Woller N. Hoenicke L. Wuestefeld T. Dauch D. (2011). Senescence surveillance of pre-malignant hepatocytes limits liver cancer development. *Nature* 479 547–551. 10.1038/nature10599 22080947

[B93] Karlseder J. Hoke K. Mirzoeva O. K. Bakkenist C. Kastan M. B. Petrini J. H. (2004). The telomeric protein TRF2 binds the ATM kinase and can inhibit the ATM-dependent DNA damage response. *PLoS Biol.* 2:E240. 10.1371/journal.pbio.0020240 15314656 PMC509302

[B94] Katakura Y. Nakata E. Miura T. Shirahata S. (1999). Transforming growth factor beta triggers two independent-senescence programs in cancer cells. *Biochem. Biophys. Res. Commun.* 255 110–115. 10.1006/bbrc.1999.0129 10082664

[B95] Khosravi-Far R. Solski P. A. Clark G. J. Kinch M. S. Der C. J. (1995). Activation of Rac1, RhoA, and mitogen-activated protein kinases is required for Ras transformation. *Mol. Cell. Biol.* 15 6443–6453. 10.1128/mcb.15.11.6443 7565796 PMC230895

[B96] Kim S. J. Lee H. W. Gu Kang H. La S. H. Choi I. J. Ro J. Y. (2014). Ablation of galectin-3 induces p27(KIP1)-dependent premature senescence without oncogenic stress. *Cell Death Differ.* 21 1769–1779. 10.1038/cdd.2014.88 24971481 PMC4211374

[B97] Kim Y. H. Choi Y. W. Lee J. Soh E. Y. Kim J. H. Park T. J. (2017). Senescent tumor cells lead the collective invasion in thyroid cancer. *Nat. Commun.* 8:15208. 10.1038/ncomms15208 PMC5436223 28489070

[B98] Kolquist K. A. Ellisen L. W. Counter C. M. Meyerson M. Tan L. K. Weinberg R. A. (1998). Expression of TERT in early premalignant lesions and a subset of cells in normal tissues. *Nat. Genet.* 19 182–186. 10.1038/554 9620778

[B99] Korkaya H. Kim G. I. Davis A. Malik F. Henry N. L. Ithimakin S. (2012). Activation of an IL6 inflammatory loop mediates trastuzumab resistance in HER2+ breast cancer by expanding the cancer stem cell population. *Mol. Cell* 47 570–584. 10.1016/j.molcel.2012.06.014 22819326 PMC3432419

[B100] Kosar M. Bartkova J. Hubackova S. Hodny Z. Lukas J. Bartek J. (2011). Senescence-associated heterochromatin foci are dispensable for cellular senescence, occur in a cell type- And insult-dependent manner, and follow expression of p16^ink4a^. *Cell Cycle (Georgetown, Tex.)* 10 457–468. 10.4161/cc.10.3.14707 21248468

[B101] Krtolica A. Parrinello S. Lockett S. Desprez P. Y. Campisi J. (2001). Senescent fibroblasts promote epithelial cell growth and tumorigenesis: a link between cancer and aging. *Proc. Natl. Acad. Sci. U.S.A.* 98 12072–12077. 10.1073/pnas.211053698 11593017 PMC59769

[B102] Kuilman T. Michaloglou C. Mooi W. J. Peeper D. S. (2010). The essence of senescence. *Genes Dev.* 24 2463–2479. 10.1101/gad.1971610 21078816 PMC2975923

[B103] Kuilman T. Michaloglou C. Vredeveld L. C. Douma S. van Doorn R. Desmet C. J. (2008). Oncogene-induced senescence relayed by an interleukin-dependent inflammatory network. *Cell* 133, 1019–1031. 10.1016/j.cell.2008.03.039 18555778

[B104] Kurz D. J. Decary S. Hong Y. Erusalimsky J. D. (2000). Senescence-associated (beta)-galactosidase reflects an increase in lysosomal mass during replicative ageing of human endothelial cells. *J. Cell Sci.* 113 (Pt 20) 3613–3622. 10.1242/jcs.113.20.3613 11017877

[B105] Laberge R. M. Sun Y. Orjalo A. V. Patil C. K. Freund A. Zhou L. (2015). MTOR regulates the pro-tumorigenic senescence-associated secretory phenotype by promoting IL1A translation. *Nat. Cell Biol.* 17 1049–1061. 10.1038/ncb3195 26147250 PMC4691706

[B106] Lasry A. Ben-Neriah Y. (2015). Senescence-associated inflammatory responses: aging and cancer perspectives. *Trends Immunol.* 36 217–228. 10.1016/j.it.2015.02.009 25801910

[B107] Lecot P. Alimirah F. Desprez P. Y. Campisi J. Wiley C. (2016). Context-dependent effects of cellular senescence in cancer development. *Br. J. Cancer* 114 1180–1184. 10.1038/bjc.2016.115 27140310 PMC4891501

[B108] Lee A. C. Fenster B. E. Ito H. Takeda K. Bae N. S. Hirai T. (1999). Ras proteins induce senescence by altering the intracellular levels of reactive oxygen species. *J. Biol. Chem.* 274 7936–7940. 10.1074/jbc.274.12.7936 10075689

[B109] Lee B. Y. Han J. A. Im J. S. Morrone A. Johung K. Goodwin E. C. (2006). Senescence-associated beta-galactosidase is lysosomal beta-galactosidase. *Aging Cell* 5 187–195. 16626397 10.1111/j.1474-9726.2006.00199.x

[B110] Lee J. J. Kim B. C. Park M. J. Lee Y. S. Kim Y. N. Lee B. L. (2011). PTEN status switches cell fate between premature senescence and apoptosis in glioma exposed to ionizing radiation. *Cell Death Diff.* 18 666–677. 10.1038/cdd.2010.139 21072054 PMC3131905

[B111] Lee J. J. Lee J. H. Ko Y. G. Hong S. I. Lee J. S. (2010). Prevention of premature senescence requires JNK regulation of Bcl-2 and reactive oxygen species. *Oncogene* 29 561–575. 10.1038/onc.2009.355 19855432

[B112] Levy M. Z. Allsopp R. C. Futcher A. B. Greider C. W. Harley C. B. (1992). Telomere end-replication problem and cell aging. *J. Mol. Biol.* 225 951–960. 10.1016/0022-2836(92)90096-3 1613801

[B113] Li F. Huangyang P. Burrows M. Guo K. Riscal R. (2020). FBP1 loss disrupts liver metabolism and promotes tumorigenesis through a hepatic stellate cell senescence secretome. *Nat. Cell Biol.* 22 728–739. 10.1038/s41556-020-0511-2 32367049 PMC7286794

[B114] Li L. Huang Y. Gao Y. Shi T. Xu Y. Li H. (2018). EGF/EGFR upregulates and cooperates with Netrin-4 to protect glioblastoma cells from DNA damage-induced senescence. *BMC Cancer* 18:1215. 10.1186/s12885-018-5056-4 30514230 PMC6280426

[B115] Lim N. Townsend P. A. (2020). Cdc6 as a novel target in cancer: oncogenic potential, senescence and subcellular localisation. *Int. J. Cancer* 147 1528–1534. 10.1002/ijc.32900 32010971 PMC7496346

[B116] Lin A. W. Barradas M. Stone J. C. van Aelst L. Serrano M. Lowe S. W. (1998). Premature senescence involving p53 and p16 is activated in response to constitutive MEK/MAPK mitogenic signaling. *Genes Dev.* 12 3008–3019. 10.1101/gad.12.19.3008 9765203 PMC317198

[B117] Liontos M. Koutsami M. Sideridou M. Evangelou K. Kletsas D. Levy B. (2007). Deregulated overexpression of hCdt1 and hCdc6 promotes malignant behavior. *Cancer Res.* 67 10899–10909. 10.1158/0008-5472.can-07-2837 18006835

[B118] Liu J. Liu Y. Chen J. Hu C. Teng M. Jiao K. (2017). The ROS-mediated activation of IL-6/STAT3 signaling pathway is involved in the 27-hydroxycholesterol-induced cellular senescence in nerve cells. *Toxicol. In Vitro Int. J. Publ. Assoc. BIBRA* 45(Pt 1) 10–18. 10.1016/j.tiv.2017.07.013 28739487

[B119] Liu S. Uppal H. Demaria M. Desprez P. Y. Campisi J. Kapahi P. (2015). Simvastatin suppresses breast cancer cell proliferation induced by senescent cells. *Sci. Rep.* 5:17895. 10.1038/srep17895 PMC4677323 26658759

[B120] Loaiza N. Demaria M. (2016). Cellular senescence and tumor promotion: Is aging the key? *Biochim. Biophys. Acta* 1865 155–167. 10.1016/j.bbcan.2016.01.007 26845683

[B121] Lou Z. Minter-Dykhouse K. Wu X. Chen J. (2003). MDC1 is coupled to activated CHK2 in mammalian DNA damage response pathways. *Nature* 421 957–961. 10.1038/nature01447 12607004

[B122] Lowe S. W. Cepero E. Evan G. (2004). Intrinsic tumour suppression. *Nature* 432 307–315. 10.1038/nature03098 15549092

[B123] Lujambio A. Akkari L. Simon J. Grace D. Tschaharganeh D. F. Bolden J. E. (2013). Non-cell-autonomous tumor suppression by p53. *Cell* 153 449–460. 10.1016/j.cell.2013.03.020 23562644 PMC3702034

[B124] Macha M. A. Rachagani S. Pai P. Gupta S. Lydiatt W. M. Smith R. B. (2015). MUC4 regulates cellular senescence in head and neck squamous cell carcinoma through p16/Rb pathway. *Oncogene* 34 1698–1708. 10.1038/onc.2014.102 24747969 PMC4205229

[B125] Maicher A. Kastner L. Dees M. Luke B. (2012). Deregulated telomere transcription causes replication-dependent telomere shortening and promotes cellular senescence. *Nucleic Acids Res.* 40 6649–6659. 10.1093/nar/gks358 22553368 PMC3413150

[B126] Malaquin N. Martinez A. Rodier F. (2016). Keeping the senescence secretome under control: molecular reins on the senescence-associated secretory phenotype. *Exp. Gerontol.* 82 39–49. 10.1016/j.exger.2016.05.010 27235851

[B127] Marino Gammazza A. Campanella C. Barone R. Caruso Bavisotto C. Gorska M. Wozniak M. (2017). Doxorubicin anti-tumor mechanisms include Hsp60 post-translational modifications leading to the Hsp60/p53 complex dissociation and instauration of replicative senescence. *Cancer Lett.* 385 75–86. 10.1016/j.canlet.2016.10.045 27836734

[B128] Martins I. Raza S. Q. Voisin L. Dakhli H. Allouch A. Law F. (2018). Anticancer chemotherapy and radiotherapy trigger both non-cell-autonomous and cell-autonomous death. *Cell Death Dis.* 9:716. 10.1038/s41419-018-0747-y PMC6006149 29915308

[B129] Medema J. P. (2018). Escape from senescence boosts tumour growth. *Nature* 553 37–38. 10.1038/d41586-017-08652-0 32086500

[B130] Meng Y. Efimova E. V. Hamzeh K. W. Darga T. E. Mauceri H. J. Fu Y. X. (2012). Radiation-inducible immunotherapy for cancer: senescent tumor cells as a cancer vaccine. *Mol. Ther, J. Am. Soc. Gene Ther.* 20 1046–1055. 10.1038/mt.2012.19 22334019 PMC3345982

[B131] Meyne J. Ratliff R. L. Moyzis R. K. (1989). Conservation of the human telomere sequence (TTAGGG)n among vertebrates. *Proc. Natl. Acad. Sci. U.S.A.* 86 7049–7053. 10.1073/pnas.86.18.7049 2780561 PMC297991

[B132] Michaloglou C. Vredeveld L. C. Soengas M. S. Denoyelle C. Kuilman T. van der Horst C. M. (2005). BRAFE600-associated senescence-like cell cycle arrest of human naevi. *Nature* 436 720–724. 10.1038/nature03890 16079850

[B133] Milanovic M. Fan D. N. Y. Belenki D. Dabritz J. H. M. Zhao Z. Yu Y. (2018). Senescence-associated reprogramming promotes cancer stemness. *Nature* 553 96–100. 10.1038/nature25167 29258294

[B134] Mo L. Zheng X. Huang H. Y. Shapiro E. Lepor H. Cordon-Cardo C. (2007). Hyperactivation of Ha-ras oncogene, but not Ink4a/Arf deficiency, triggers bladder tumorigenesis. *J. Clin. Invest.* 117 314–325. 10.1172/jci30062 17256055 PMC1770948

[B135] Mongiardi M. P. Radice G. Piras M. Stagni V. Pacioni S. Re A. (2019). Axitinib exposure triggers endothelial cells senescence through ROS accumulation and ATM activation. *Oncogene* 38 5413–5424. 10.1038/s41388-019-0798-2 30967634

[B136] Morelli M. B. Amantini C. Nabissi M. Cardinali C. Santoni M. Bernardini G. (2017). Axitinib induces senescence-associated cell death and necrosis in glioma cell lines: The proteasome inhibitor, bortezomib, potentiates axitinib-induced cytotoxicity in a p21(Waf/Cip1) dependent manner. *Oncotarget* 8 3380–3395. 10.18632/oncotarget.13769 27926485 PMC5356889

[B137] Mosteiro L. Pantoja C. Alcazar N. Marion R. M. Chondronasiou D. Rovira M. (2016). Tissue damage and senescence provide critical signals for cellular reprogramming *in vivo*. *Science (New York, N.Y.)* 354:aaf4445. 10.1126/science.aaf4445 27884981

[B138] Mosteiro L. Pantoja C. de Martino A. Serrano M. (2018). Senescence promotes in vivo reprogramming through p16(INK)(4a) and IL-6. *Aging Cell* 17:e12711. 10.1111/acel.12711 29280266 PMC5847859

[B139] Mudbhary R. Hoshida Y. Chernyavskaya Y. Jacob V. Villanueva A. Fiel M. I. (2014). UHRF1 overexpression drives DNA hypomethylation and hepatocellular carcinoma. *Cancer Cell* 25 196–209. 10.1016/j.ccr.2014.01.003 24486181 PMC3951208

[B140] Nacarelli T. Fukumoto T. Zundell J. A. Fatkhutdinov N. Jean S. Cadungog M. G. (2020). NAMPT inhibition suppresses cancer stem-like cells associated with therapy-induced senescence in ovarian cancer. *Cancer Res.* 80 890–900. 10.1158/0008-5472.can-19-2830 31857293 PMC7024650

[B141] Nakamura A. J. Chiang Y. J. Hathcock K. S. Horikawa I. Sedelnikova O. A. Hodes R. J. (2008). Both telomeric and non-telomeric DNA damage are determinants of mammalian cellular senescence. *Epigenetics Chromatin* 1:6. 10.1186/1756-8935-1-6 PMC2584625 19014415

[B142] Nardella C. Chen Z. Salmena L. Carracedo A. Alimonti A. Egia A. (2008). Aberrant Rheb-mediated mTORC1 activation and Pten haploinsufficiency are cooperative oncogenic events. *Genes Dev.* 22 2172–2177. 10.1101/gad.1699608 18708577 PMC2518820

[B143] Narita M. Narita M. Krizhanovsky V. Nunez S. Chicas A. Hearn S. A. (2006). A novel role for high-mobility group a proteins in cellular senescence and heterochromatin formation. *Cell* 126 503–514. 10.1016/j.cell.2006.05.052 16901784

[B144] Narita M. Nunez S. Heard E. Narita M. Lin A. W. Hearn S. A. (2003). Rb-mediated heterochromatin formation and silencing of E2F target genes during cellular senescence. *Cell* 113 703–716. 10.1016/s0092-8674(03)00401-x 12809602

[B145] Nekulova M. Holcakova J. Coates P. Vojtesek B. (2011). The role of p63 in cancer, stem cells and cancer stem cells. *Cell. Mol. Biol. Lett.* 16 296–327. 21442444 10.2478/s11658-011-0009-9 PMC6275999

[B146] Noguchi S. Mori T. Otsuka Y. Yamada N. Yasui Y. Iwasaki J. (2012). Anti-oncogenic microRNA-203 induces senescence by targeting E2F3 protein in human melanoma cells. *J. Biol. Chem.* 287 11769–11777. 10.1074/jbc.m111.325027 22354972 PMC3320925

[B147] Nowicki T. S. Zhao H. Darzynkiewicz Z. Moscatello A. Shin E. Schantz S. (2011). Downregulation of uPAR inhibits migration, invasion, proliferation, FAK/PI3K/Akt signaling and induces senescence in papillary thyroid carcinoma cells. *Cell Cycle (Georgetown, Tex.)* 10 100–107. 10.4161/cc.10.1.14362 21191179 PMC3048079

[B148] Ohashi S. Natsuizaka M. Wong G. S. Michaylira C. Z. Grugan K. D. Stairs D. B. (2010). Epidermal growth factor receptor and mutant p53 expand an esophageal cellular subpopulation capable of epithelial-to-mesenchymal transition through ZEB transcription factors. *Cancer Res.* 70 4174–4184. 10.1158/0008-5472.can-09-4614 20424117 PMC3007622

[B149] Ohtani N. Yamakoshi K. Takahashi A. Hara E. (2004). The p16INK4a-RB pathway: molecular link between cellular senescence and tumor suppression. *J. Med. Invest. JMI* 51 146–153. 10.2152/jmi.51.146 15460900

[B150] Orjalo A. V. Bhaumik D. Gengler B. K. Scott G. K. Campisi J. (2009). Cell surface-bound IL-1alpha is an upstream regulator of the senescence-associated IL-6/IL-8 cytokine network. *Proc. Natl. Acad. Sci. U.S.A.* 106 17031–17036. 10.1073/pnas.0905299106 19805069 PMC2761322

[B151] Osman A. A. Monroe M. M. Ortega Alves M. V. Patel A. A. Katsonis P. Fitzgerald A. L. (2015). Wee-1 kinase inhibition overcomes cisplatin resistance associated with high-risk TP53 mutations in head and neck cancer through mitotic arrest followed by senescence. *Mol. Cancer Therap.* 14 608–619. 10.1158/1535-7163.mct-14-0735-t 25504633 PMC4557970

[B152] Oyama K. Okawa T. Nakagawa H. Takaoka M. Andl C. D. Kim S. H. (2007). AKT induces senescence in primary esophageal epithelial cells but is permissive for differentiation as revealed in organotypic culture. *Oncogene* 26 2353–2364. 10.1038/sj.onc.1210025 17043653 PMC2996093

[B153] Park G. B. Jeong J. Y. (2019). Gliotoxin enhances autophagic cell death via the DAPK1-TAp63 signaling pathway in paclitaxel-resistant ovarian cancer cells. *Mar. Drugs* 17:412. 10.3390/md17070412 31336860 PMC6669733

[B154] Parrinello S. Samper E. Krtolica A. Goldstein J. Melov S. Campisi J. (2003). Oxygen sensitivity severely limits the replicative lifespan of murine fibroblasts. *Nat. Cell Biol.* 5 741–747. 10.1038/ncb1024 12855956 PMC4940195

[B155] Passos J. F. Nelson G. Wang C. Richter T. Simillion C. Proctor C. J. (2010). Feedback between p21 and reactive oxygen production is necessary for cell senescence. *Mol. Syst. Biol.* 6:347. 10.1038/msb.2010.5 20160708 PMC2835567

[B156] Patten D. A. Lee S. G. Parks R. J. Chan D. W. Harper M. E. Tsang B. K. (2019). Jumonji domain-containing 6 (JMJD6) identified as a potential therapeutic target in ovarian cancer. *Mol. Carcinog.* 4:24. 10.1038/s41392-019-0055-8 PMC6799828 31637004

[B157] Peeper D. S. (2011). Ageing: old cells under attack. *Nature* 479 186–187. 10.1038/479186a 22071762

[B158] Perez-Campo F. M. Costa G. Lie A. L. M. Stifani S. Kouskoff V. Lacaud G. (2014). MOZ-mediated repression of p16(INK) (4) (a) is critical for the self-renewal of neural and hematopoietic stem cells. *Stem Cells (Dayton, Ohio)* 32 1591–1601. 10.1002/stem.1606 24307508 PMC4237135

[B159] Perez-Mancera P. A. Young A. R. Narita M. (2014). Inside and out: the activities of senescence in cancer. *Nat. Rev. Cancer* 14 547–558. 10.1038/nrc3773 25030953

[B160] Pernicova Z. Slabakova E. Kharaishvili G. Bouchal J. Kral M. Kunicka Z. (2011). Androgen depletion induces senescence in prostate cancer cells through down-regulation of Skp2. *Neoplasia (New York, N.Y.)* 13 526–536. 10.1593/neo.11182 21677876 PMC3114246

[B161] Pomerantz J. Schreiber-Agus N. Liegeois N. J. Silverman A. Alland L. Chin L. (1998). The Ink4a tumor suppressor gene product, p19Arf, interacts with MDM2 and neutralizes MDM2’s inhibition of p53. *Cell* 92 713–723. 10.1016/s0092-8674(00)81400-2 9529248

[B162] Pribluda A. Elyada E. Wiener Z. Hamza H. Goldstein R. E. Biton M. (2013). A senescence-inflammatory switch from cancer-inhibitory to cancer-promoting mechanism. *Cancer Cell* 24 242–256. 10.1016/j.ccr.2013.06.005 23890787

[B163] Qian Y. Zhang J. Yan B. Chen X. (2008). DEC1, a basic helix-loop-helix transcription factor and a novel target gene of the p53 family, mediates p53-dependent premature senescence. *J. Biol. Chem.* 283 2896–2905. 10.1074/jbc.m708624200 18025081 PMC4118587

[B164] Quelle D. E. Zindy F. Ashmun R. A. Sherr C. J. (1995). Alternative reading frames of the INK4a tumor suppressor gene encode two unrelated proteins capable of inducing cell cycle arrest. *Cell* 83 993–1000. 10.1016/0092-8674(95)90214-7 8521522

[B165] Rao S. G. Jackson J. G. (2016). SASP: tumor suppressor or promoter? Yes! *Trends Cancer* 2 676–687. 10.1016/j.trecan.2016.10.001 28741506

[B166] Ren X. S. Yin M. H. Zhang X. Wang Z. Feng S. P. Wang G. X. (2014). Tumor-suppressive microRNA-449a induces growth arrest and senescence by targeting E2F3 in human lung cancer cells. *Cancer Lett.* 344 195–203. 10.1016/j.canlet.2013.10.031 24211326

[B167] Revandkar A. Perciato M. L. Toso A. Alajati A. Chen J. Gerber H. (2016). Inhibition of Notch pathway arrests PTEN-deficient advanced prostate cancer by triggering p27-driven cellular senescence. *Nat. Commun.* 7:13719. 10.1038/ncomms13719 PMC5159884 27941799

[B168] Ritschka B. Storer M. Mas A. Heinzmann F. Ortells M. C. Morton J. P. (2017). The senescence-associated secretory phenotype induces cellular plasticity and tissue regeneration. *Genes Dev.* 31 172–183. 10.1101/gad.290635.116 28143833 PMC5322731

[B169] Robinson A. R. Yousefzadeh M. J. Rozgaja T. A. Wang J. Li X. Tilstra J. S. (2018). Spontaneous DNA damage to the nuclear genome promotes senescence, redox imbalance and aging. *Redox Biol.* 17 259–273. 10.1016/j.redox.2018.04.007 29747066 PMC6006678

[B170] Robles S. J. Adami G. R. (1998). Agents that cause DNA double strand breaks lead to p16INK4a enrichment and the premature senescence of normal fibroblasts. *Oncogene* 16 1113–1123. 10.1038/sj.onc.1201862 9528853

[B171] Rodier F. Campisi J. (2011). Four faces of cellular senescence. *J. Cell Biol.* 192 547–556. 10.1083/jcb.201009094 21321098 PMC3044123

[B172] Rodier F. Coppe J. P. Patil C. K. Hoeijmakers W. A. Munoz D. P. Raza S. R. (2009). Persistent DNA damage signalling triggers senescence-associated inflammatory cytokine secretion. *Nat. Cell Biol.* 11 973–979. 10.1038/ncb1909 19597488 PMC2743561

[B173] Rodier F. Munoz D. P. Teachenor R. Chu V. Le O. Bhaumik D. (2011). DNA-SCARS: distinct nuclear structures that sustain damage-induced senescence growth arrest and inflammatory cytokine secretion. *J. Cell Sci.* 124(Pt 1) 68–81. 10.1242/jcs.071340 21118958 PMC3001408

[B174] Roediger J. Lornez V. Kyrylenko S. Baniahmad A. (2010). Histone deacetylase inhibitors induce cellular senescence in neuroblastoma and prostate cancer. *Medizinische Genetik* 22:178.

[B175] Romagosa C. Simonetti S. Lopez-Vicente L. Mazo A. Lleonart M. E. Castellvi J. (2011). p16(Ink4a) overexpression in cancer: a tumor suppressor gene associated with senescence and high-grade tumors. *Oncogene* 30 2087–2097. 10.1038/onc.2010.614 21297668

[B176] Rufini A. Tucci P. Celardo I. Melino G. (2013). Senescence and aging: the critical roles of p53. *Oncogene* 32 5129–5143. 10.1038/onc.2012.640 23416979

[B177] Ruhland M. K. Loza A. J. Capietto A. H. (2016). Stromal senescence establishes an immunosuppressive microenvironment that drives tumorigenesis. *Nat. Commun.* 7:11762. 10.1038/ncomms11762 PMC4899869 27272654

[B178] Ruscetti M. Morris J.P.t Mezzadra R. Russell J. Leibold J. Romesser P. B. (2020). Senescence-induced vascular remodeling creates therapeutic vulnerabilities in pancreas cancer. *Cell* 181 424–441.e21. 32234521 10.1016/j.cell.2020.03.008 PMC7278897

[B179] Sai X. Qin C. Wu Y. Zhao Y. Bian T. (2020). Downregulation of PTEN mediates bleomycin-induced premature senescence in lung cancer cells by suppressing autophagy. *J. Int. Med. Res.* 48:300060520923522. 10.1177/0300060520923522 PMC7287201 32436415

[B180] Schleich K. Kase J. Dörr J. R. Trescher S. Bhattacharya A. Yu Y. (2020). H3K9me3-mediated epigenetic regulation of senescence in mice predicts outcome of lymphoma patients. *Nat. Commun.* 11:3651. 10.1038/s41467-020-17467-z PMC7371731 32686676

[B181] Sedelnikova O. A. Horikawa I. Zimonjic D. B. Popescu N. C. Bonner W. M. Barrett J. C. (2004). Senescing human cells and ageing mice accumulate DNA lesions with unrepairable double-strand breaks. *Nat. Cell Biol.* 6 168–170. 10.1038/ncb1095 14755273

[B182] Seo S. B. Baek J. Y. Lim J. H. Jin X. Lee M. Y. Lee J. H. (2019). 14-3-3ζ targeting induced senescence in Hep-2 laryngeal cancer cell through deneddylation of Cullin1 in the Skp1-Cullin-F-box protein complex. *Cell Proliferation* 52:e12654. 10.1111/cpr.12654 PMC6797561 31222857

[B183] Serrano M. Hannon G. J. Beach D. (1993). A new regulatory motif in cell-cycle control causing specific inhibition of cyclin D/CDK4. *Nature* 366 704–707. 10.1038/366704a0 8259215

[B184] Serrano M. Lin A. W. McCurrach M. E. Beach D. Lowe S. W. (1997). Oncogenic ras provokes premature cell senescence associated with accumulation of p53 and p16INK4a. *Cell* 88 593–602. 10.1016/s0092-8674(00)81902-9 9054499

[B185] Sharpless N. E. Sherr C. J. (2015). Forging a signature of in vivo senescence. *Nat. Rev. Cancer* 15 397–408. 10.1038/nrc3960 26105537

[B186] Sheikh B. N. Phipson B. El-Saafin F. Vanyai H. K. Downer N. L. Bird M. J. (2015). MOZ (MYST3, KAT6A) inhibits senescence via the INK4A-ARF pathway. *Oncogene* 34 5807–5820. 10.1038/onc.2015.33 25772242

[B187] Shiloh Y. (2003). ATM and related protein kinases: safeguarding genome integrity. *Nat. Rev. Cancer* 3 155–168. 10.1038/nrc1011 12612651

[B188] Shiloh Y. (2006). The ATM-mediated DNA-damage response: taking shape. *Trends Biochem. Sci.* 31 402–410. 10.1016/j.tibs.2006.05.004 16774833

[B189] Song J. H. Lee C. J. An H. J. Yoo S. M. Kang H. C. Lee J. Y. (2019). Magnolin targeting of ERK1/2 inhibits cell proliferation and colony growth by induction of cellular senescence in ovarian cancer cells. *Mol. Carcinog.* 58 88–101. 10.1002/mc.22909 30230030 PMC6585859

[B190] Soucek L. Whitfield J. Martins C. P. Finch A. J. Murphy D. J. Sodir N. M. (2008). Modelling Myc inhibition as a cancer therapy. *Nature* 455 679–683. 18716624 10.1038/nature07260 PMC4485609

[B191] Stein G. H. Drullinger L. F. Soulard A. Dulic V. (1999). Differential roles for cyclin-dependent kinase inhibitors p21 and p16 in the mechanisms of senescence and differentiation in human fibroblasts. *Mol. Cell. Biol.* 19 2109–2117. 10.1128/mcb.19.3.2109 10022898 PMC84004

[B192] Stewart S. A. Ben-Porath I. Carey V. J. O’Connor B. F. Hahn W. C. Weinberg R. A. (2003). Erosion of the telomeric single-strand overhang at replicative senescence. *Nat. Genet.* 33 492–496. 10.1038/ng1127 12652299

[B193] Storer M. Mas A. Robert-Moreno A. Pecoraro M. Ortells M. C. Di Giacomo V. (2013). XSenescence is a developmental mechanism that contributes to embryonic growth and patterning. *Cell* 155 X1119–X1130. 10.1016/j.cell.2013.10.041 24238961

[B194] Sun C. Wang L. Huang S. Heynen G. J. Prahallad A. Robert C. (2014). Reversible and adaptive resistance to BRAF(V600E) inhibition in melanoma. *Nature* 508 118–122. 10.1038/nature13121 24670642

[B195] Sun H. Wang H. Wang X. Aoki Y. Wang X. Yang Y. (2020). Aurora-A/SOX8/FOXK1 signaling axis promotes chemoresistance via suppression of cell senescence and induction of glucose metabolism in ovarian cancer organoids and cells. *Theranostics* 10 6928–6945. 10.7150/thno.43811 32550913 PMC7295065

[B196] Sun P. Yoshizuka N. New L. Moser B. A. Li Y. Liao R. (2007). PRAK is essential for ras-induced senescence and tumor suppression. *Cell* 128 295–308. 10.1016/j.cell.2006.11.050 17254968

[B197] Sun X. Shi B. Zheng H. Min L. Yang J. Li X. (2018). Senescence-associated secretory factors induced by cisplatin in melanoma cells promote non-senescent melanoma cell growth through activation of the ERK1/2-RSK1 pathway. *Cell Death Dis.* 9:260. 10.1038/s41419-018-0303-9 PMC5833767 29449532

[B198] Takai H. Smogorzewska A. de Lange T. (2003). DNA damage foci at dysfunctional telomeres. *Curr. Biol. CB* 13 1549–1556. 10.1016/s0960-9822(03)00542-6 12956959

[B199] Takasugi M. Okada R. Takahashi A. Virya Chen D. (2017). Small extracellular vesicles secreted from senescent cells promote cancer cell proliferation through EphA2. *Nat. Commun.* 8:15729. 10.1038/ncomms15728 PMC5467215 28585531

[B200] Taschner-Mandl S. Schwarz M. Blaha J. Kauer M. Kromp F. Frank N. (2016). Metronomic topotecan impedes tumor growth of MYCN-amplified neuroblastoma cells in vitro and in vivo by therapy induced senescence. *Oncotarget* 7 3571–3586. 10.18632/oncotarget.6527 26657295 PMC4823128

[B201] Tasdemir N. Banito A. Roe J. S. Alonso-Curbelo D. Camiolo M. Tschaharganeh D. F. (2016). BRD4 connects enhancer remodeling to senescence immune surveillance. *Cancer Discov.* 6 612–629. 10.1158/2159-8290.cd-16-0217 27099234 PMC4893996

[B202] Tivari S. Lu H. Dasgupta T. De Lorenzo M. S. Wieder R. (2018). Reawakening of dormant estrogen-dependent human breast cancer cells by bone marrow stroma secretory senescence. *Cell Commun. Signal.* 16:48. 10.1186/s12964-018-0259-5 PMC6098600 30119678

[B203] Urano T. Emkey R. Feig L. A. (1996). Ral-GTPases mediate a distinct downstream signaling pathway from Ras that facilitates cellular transformation. *EMBO J.* 15 810–816. 10.1002/j.1460-2075.1996.tb00416.x 8631302 PMC450279

[B204] van Deursen J. M. (2014). The role of senescent cells in ageing. *Nature* 509 439–446. 10.1038/nature13193 24848057 PMC4214092

[B205] Vaziri H. Benchimol S. (1998). Reconstitution of telomerase activity in normal human cells leads to elongation of telomeres and extended replicative life span. *Curr. Biol. CB* 8 279–282. 10.1016/s0960-9822(98)70109-5 9501072

[B206] Ventura A. Kirsch D. G. McLaughlin M. E. Tuveson D. A. Grimm J. Lintault L. (2007). Restoration of p53 function leads to tumour regression in vivo. *Nature* 445 661–665. 10.1038/nature05541 17251932

[B207] Verdun R. E. Crabbe L. Haggblom C. Karlseder J. (2005). Functional human telomeres are recognized as DNA damage in G2 of the cell cycle. *Mol. Cell* 20 551–561. 10.1016/j.molcel.2005.09.024 16307919

[B208] von Zglinicki T. Martin-Ruiz C. M. (2005). Telomeres as biomarkers for ageing and age-related diseases. *Curr. Mol. Med.* 5 197–203. 10.2174/1566524053586545 15974873

[B209] Vredeveld L. C. Possik P. A. Smit M. A. Meissl K. Michaloglou C. Horlings H. M. (2012). Abrogation of BRAFV600E-induced senescence by PI3K pathway activation contributes to melanomagenesis. *Genes Dev.* 26 1055–1069. 10.1101/gad.187252.112 22549727 PMC3360561

[B210] Wagner V. Gil J. (2020). Senescence as a therapeutically relevant response to CDK4/6 inhibitors. *Oncogene* 39 5165–5176. 10.1038/s41388-020-1354-9 32541838 PMC7610384

[B211] Wang C. Jurk D. Maddick M. Nelson G. Martin-Ruiz C. von Zglinicki T. (2009). DNA damage response and cellular senescence in tissues of aging mice. *Aging Cell* 8 311–323. 10.1111/j.1474-9726.2009.00481.x 19627270

[B212] Wang M. Morsbach F. Sander D. Gheorghiu L. Nanda A. Benes C. (2011). EGF receptor inhibition radiosensitizes NSCLC cells by inducing senescence in cells sustaining DNA double-strand breaks. *Cancer Res.* 71 6261–6269. 10.1158/0008-5472.can-11-0213 21852385 PMC3185115

[B213] Wang T. Notta F. Navab R. Joseph J. Ibrahimov E. Xu J. (2017). Senescent carcinoma-associated fibroblasts upregulate IL8 to enhance prometastatic phenotypes. *Mol. Cancer Res. MCR* 15 3–14. 10.1158/1541-7786.mcr-16-0192 27678171

[B214] Wang Z. Gao J. (2019). Olaparib induced senescence under P16 or P53 dependent manner in ovarian cancer. *J. Gynecol. Oncol.* 30:e26. 10.3802/jgo.2019.30.e26 PMC6393639 30740957

[B215] Wang Z. Li Y. Wu D. Yu S. Wang Y. Leung Chan F. (2020). Nuclear receptor HNF4α performs a tumor suppressor function in prostate cancer via its induction of p21-driven cellular senescence. *Oncogene* 39 1572–1589. 10.1038/s41388-019-1080-3 31695151 PMC7018660

[B216] Wang Z. Ma L. Su M. Zhou Y. Mao K. Li C. (2018). Baicalin induces cellular senescence in human colon cancer cells via upregulation of DEPP and the activation of Ras/Raf/MEK/ERK signaling. *Cell Death Dis.* 9:217. 10.1038/s41419-017-0223-0 PMC5833439 29440765

[B217] Wei S. Wei S. Sedivy J. M. (1999). Expression of catalytically active telomerase does not prevent premature senescence caused by overexpression of oncogenic Ha-Ras in normal human fibroblasts. *Cancer Res.* 59 1539–1543. 10197626

[B218] Wei W. Herbig U. Wei S. Dutriaux A. Sedivy J. M. (2003). Loss of retinoblastoma but not p16 function allows bypass of replicative senescence in human fibroblasts. *EMBO Rep.* 4 1061–1066. 10.1038/sj.embor.7400001 14566323 PMC1326371

[B219] Weiner-Gorzel K. Dempsey E. Milewska M. McGoldrick A. Toh V. Walsh A. (2015). Overexpression of the microRNA miR-433 promotes resistance to paclitaxel through the induction of cellular senescence in ovarian cancer cells. *Cancer Med.* 4 745–758. 10.1002/cam4.409 25684390 PMC4430267

[B220] White M. A. Nicolette C. Minden A. Polverino A. Van Aelst L. Karin M. (1995). Multiple Ras functions can contribute to mammalian cell transformation. *Cell* 80 533–541. 10.1016/0092-8674(95)90507-3 7867061

[B221] Widemann B. C. Italiano A. (2018). Biology and management of undifferentiated pleomorphic sarcoma, myxofibrosarcoma, and malignant peripheral nerve sheath tumors: state of the art and perspectives. *J. Clin. Oncol. J. Am. Soc. Clin. Oncol.* 36 160–167. 10.1200/jco.2017.75.3467 29220302 PMC5759316

[B222] Wiley C. D. (2020). Bubble bubble, senescent cells are a cauldron of tumor trouble. *Cancer Res.* 80 3193–3194. 10.1158/0008-5472.can-20-1811 32817014

[B223] Wright W. E. Tesmer V. M. Huffman K. E. Levene S. D. Shay J. W. (1997). Normal human chromosomes have long G-rich telomeric overhangs at one end. *Genes Dev.* 11 2801–2809. 10.1101/gad.11.21.2801 9353250 PMC316649

[B224] Wu C. H. van Riggelen J. Yetil A. Fan A. C. Bachireddy P. Felsher D. W. (2007). Cellular senescence is an important mechanism of tumor regression upon c-Myc inactivation. *Proc. Natl. Acad. Sci. U.S.A.* 104 13028–13033. 10.1073/pnas.0701953104 17664422 PMC1941831

[B225] Wu J. Su H. K. Yu Z. H. Xi S. Y. Guo C. C. Hu Z. Y. (2020b). Skp2 modulates proliferation, senescence and tumorigenesis of glioma. *Cancer Cell Int.* 20:71. 10.1186/s12935-020-1144-z PMC7059397 32165861

[B226] Wu Q. Li B. Liu L. Sun S. Sun S. (2020a). Centrosome dysfunction: a link between senescence and tumor immunity. *Signal Trans. Targeted Ther.* 5:107. 10.1038/s41392-020-00214-7 PMC7327052 32606370

[B227] Xing R. Li W. Cui J. Zhang J. Kang B. Wang Y. (2012). Gastrokine 1 induces senescence through p16/Rb pathway activation in gastric cancer cells. *Gut* 61 43–52. 10.1136/gut.2010.230623 21676900

[B228] Xu G. Chapman J. R. Brandsma I. Yuan J. Mistrik M. Bouwman P. (2015). Rottenberg. REV7 counteracts DNA double-strand break resection and affects PARP inhibition. *Nature* 521 541–544. 10.1038/nature14328 25799992 PMC4671316

[B229] Xu H. Lin F. Wang Z. Yang L. Meng J. Ou Z. (2018). CXCR2 promotes breast cancer metastasis and chemoresistance via suppression of AKT1 and activation of COX2. *Cancer Lett.* 412 69–80. 10.1016/j.canlet.2017.09.030 28964785

[B230] Xue W. Zender L. Miething C. Dickins R. A. Hernando E. Krizhanovsky V. (2007). Senescence and tumour clearance is triggered by p53 restoration in murine liver carcinomas. *Nature* 445 656–660. 10.1038/nature05529 17251933 PMC4601097

[B231] Yaglom J. A. Gabai V. L. Sherman M. Y. (2007). High levels of heat shock protein Hsp72 in cancer cells suppress default senescence pathways. *Cancer Res.* 67 2373–2381. 10.1158/0008-5472.can-06-3796 17332370

[B232] Yamakoshi K. Takahashi A. Hirota F. Nakayama R. Ishimaru N. Kubo Y. (2009). Real-time in vivo imaging of p16Ink4a reveals cross talk with p53. *J. Cell Biol.* 186 393–407. 10.1083/jcb.200904105 19667129 PMC2728398

[B233] Yang G. J. Ko C. N. Zhong H. J. Leung C. H. Ma D. L. (2019). Structure-based discovery of a selective KDM5A inhibitor that exhibits anti-cancer activity via inducing cell cycle arrest and senescence in breast cancer cell lines. *Cancers* 11:92. 10.3390/cancers11010092 30650517 PMC6360022

[B234] Yang S. Zhao X. Wei X. Yi Y. Tsai S. H. Cheng J. C. (2017). APELA promotes tumour growth and cell migration in ovarian cancer in a p53-dependent manner. *Signal Trans. Targeted Ther.* 147 663–671. 10.1016/j.ygyno.2017.10.016 29079036

[B235] Young A. P. Schlisio S. Minamishima Y. A. Zhang Q. Li L. Grisanzio C. (2008). VHL loss actuates a HIF-independent senescence programme mediated by Rb and p400. *Nat. Cell Biol.* 10 361–369. 10.1038/ncb1699 18297059

[B236] Yu X. Liu Y. Yin L. Peng Y. Peng Y. Gao Y. (2019). Radiation-promoted CDC6 protein stability contributes to radioresistance by regulating senescence and epithelial to mesenchymal transition. *Oncogene* 38 549–563. 10.1038/s41388-018-0460-4 30158672 PMC6345673

[B237] Zacarias-Fluck M. F. Morancho B. Vicario R. Luque Garcia A. Escorihuela M. Villanueva J. (2015). Effect of cellular senescence on the growth of HER2-positive breast cancers. *J. Natl. Cancer Inst.* 107:djv020. 10.1093/jnci/djv020 25972601

[B238] Zhang L. Cheng F. Wei Y. Zhang L. Guo D. Wang B. (2019). Inhibition of TAZ contributes radiation-induced senescence and growth arrest in glioma cells. *Oncogene* 38 2788–2799. 10.1038/s41388-018-0626-0 30542117 PMC6461515

[B239] Zhang Y. Xiong Y. Yarbrough W. G. (1998). ARF promotes MDM2 degradation and stabilizes p53: ARF-INK4a locus deletion impairs both the Rb and p53 tumor suppression pathways. *Cell* 92 725–734. 10.1016/s0092-8674(00)81401-4 9529249

[B240] Zhu J. Wang H. Bishop J. M. Blackburn E. H. (1999). Telomerase extends the lifespan of virus-transformed human cells without net telomere lengthening. *Proc. Natl. Acad. Sci. U.S.A.* 96 3723–3728. 10.1073/pnas.96.7.3723 10097104 PMC22361

[B241] Zhu J. Woods D. McMahon M. Bishop J. M. (1998). Senescence of human fibroblasts induced by oncogenic Raf. *Genes Dev.* 12 2997–3007. 10.1101/gad.12.19.2997 9765202 PMC317194

[B242] Zinke J. Schneider F. T. Harter P. N. Thom S. Ziegler N. Toftgard R. (2015). beta-Catenin-Gli1 interaction regulates proliferation and tumor growth in medulloblastoma. *Mol. Cancer* 14:17. 10.1186/s12943-015-0294-4 25645196 PMC4320815

[B243] Zlotnik A. (2004). Chemokines in neoplastic progression. *Sem. Cancer Biol.* 14 181–185. 10.1016/j.semcancer.2003.10.004 15246053

[B244] Zou L. (2007). Single- and double-stranded DNA: building a trigger of ATR-mediated DNA damage response. *Genes Dev.* 21 879–885. 10.1101/gad.1550307 17437994

