# Corrigendum: The Paradoxical Role of Cellular Senescence in Cancer

**DOI:** 10.3389/fcell.2021.759761

**Published:** 2021-09-23

**Authors:** Jing Yang, Mengmeng Liu, Dongchun Hong, Musheng Zeng, Xing Zhang

**Affiliations:** ^1^Melanoma and Sarcoma Medical Oncology Unit, State Key Laboratory of Oncology in South China, Collaborative Innovation Center for Cancer Medicine, Sun Yat-sen University Cancer Center, Guangzhou, China; ^2^State Key Laboratory of Oncology in South China, Collaborative Innovation Center for Cancer Medicine, Sun Yat-sen University Cancer Center, Guangzhou, China

**Keywords:** cancer, senescence, aging, senescence-associated secretory phenotype, senescent cell

In the original article in the first sentence of **Introduction**, Paragraph 1 there were incorrect citations of “Collado et al., 2007” and “Loaiza and Demaria, 2016”. It should instead be “Hayflick and Moorhead, 1961” and “Hayflick, 1965”. The correct paragraph appears as below.

“The cellular senescence was first described by Hayflick and colleagues in which they observed that after serial cultivation *in vitro*, normal human fibroblasts exhausted their capacity to divide and entered a state of irreversible growth arrest, whereas cancer cells did not enter this growth arrest state (Hayflick and Moorhead, 1961; Hayflick, 1965).”

The authors apologize for this error and state that this does not change the scientific conclusions of the article in any way. The original article has been updated.

## Publisher's Note

All claims expressed in this article are solely those of the authors and do not necessarily represent those of their affiliated organizations, or those of the publisher, the editors and the reviewers. Any product that may be evaluated in this article, or claim that may be made by its manufacturer, is not guaranteed or endorsed by the publisher.
